# Exploring the Interplay between Metabolism and Tumor Microenvironment Based on Four Major Metabolism Pathways in Colon Adenocarcinoma

**DOI:** 10.1155/2022/2159794

**Published:** 2022-06-14

**Authors:** Xiaofang Qiao, Guangmei Zhang, Yajie Xiao, Xiaoli Cui, Zhikun Zhao, Dongfang Wu, Xuefei Liu

**Affiliations:** ^1^Department of Gastrointestinal Surgery, The Second Hospital of Jilin University, Changchun 220100, China; ^2^Department of Internal Medicine, Jilin Cancer Hospital, Jilin 220200, China; ^3^Department of Medicine, YuceBio Technology Co., Ltd., Shenzhen 440300, China; ^4^Department of Oncology, Northern Theater Command General Hospital, Shenyang 210100, China

## Abstract

Tumor metabolism plays a critical role in tumor progression. However, the interaction between metabolism and tumor microenvironment (TME) has not been comprehensively revealed in colon adenocarcinoma (COAD). We used unsupervised consensus clustering to establish three molecular subtypes (clusters) based on the enrichment score of four major metabolism pathways in TCGA-COAD dataset. GSE17536 was used as a validation dataset. Single-cell RNA sequencing data (GSE161277) was employed to further verify the reliability of subtyping and characterize the correlation between metabolism and TME. Three clusters were identified and they performed distinct prognosis and molecular features. Clust3 had the worst overall survival and the highest enrichment score of glycolysis. 86 differentially expressed genes (DEGs) were identified, in which 11 DEGs were associated with favorable prognosis and 75 DEGs were associated with poor prognosis. Striking correlations were observed between hypoxia and glycolysis, clust3 and hypoxia, and clust3 and angiogenesis (*P* < 0.001).We constructed a molecular subtyping system which was effective and reliable for predicting COAD prognosis. The 86 identified key DEGs may be greatly involved in COAD progression, and they provide new perspectives and directions for further understanding the mechanism of metabolism in promoting aggressive phenotype by interacting with TME.

## 1. Introduction

Colon adenocarcinoma (COAD) is one of the most diagnosed cancer types worldwide, which represented 6.0% of new cancer cases and was the cause of 5.8% of new cancer deaths in 2020 according to global cancer statistics [1]. Risk factors of COAD are various, which can be generally classified into unhealthy lifestyles (such as smoking, heavy alcohol, and unhealthy diet), disease-caused or drug-induced (such as long-term androgen deprivation therapy, diabetes mellitus and insulin resistance, acromegaly, and renal transplantation under long-term immunosuppression), and family history [2]. Most of COAD individuals are sporadic, about 70%, and ages above 50 years are common [3]. Although screening programs such as colonoscopy, fecal immunochemical test (FIT), and computed tomography (CT) colonography are recommended and popularized for high-risk populations in the recent decades, efficient and personalized therapeutics are still current challenges especially for metastatic patients.

Immunotherapy or immune checkpoint blockade raises the hope for metastatic patients. Targeted therapies such as vascular endothelial growth factor (VEGF) inhibitors and epidermal growth factor receptor (EGFR) inhibitors are examined to effectively hinder angiogenesis and thus impede tumor growth [4]. Immunotherapy and targeted therapy regulate tumor microenvironment (TME) and inhibit tumor progression through targeting key proteins associated with poor prognosis. Tumor metabolism is a pivotal factor in TME modulation, where four critical metabolism pathways, glycolysis, pentose phosphate pathway (PPP), fatty acid oxidation (FAO), and glutaminolysis, are reported to be strikingly involved in tumor metabolic reprogramming contributing to TME modulation and tumor progression [5–9]. Particularly, a number of glycolysis-related prognostic biomarkers have been identified in various cancer types [10–13]. Nevertheless, the mechanism of the interaction between TME and metabolism pathways has not been well understood.

An increasing number of bioinformatics tools accelerate the development of understanding potential mechanisms of cancer development and the novel prognostic biomarkers for predicting cancer prognosis [14–17]. Moreover, the development of single-cell RNA sequencing (scRNA-seq) technology allows more accurately parsing the interplay or crosstalk of various pathways in TME. In this study, we sought to reveal the interaction between metabolism pathways and TME based on expression data and single-cell data gleaned from public database. Three molecular subtypes (clusters) were established based on the expression of genes within metabolism pathways. Three clusters showed distinct overall survival and expression patterns of metabolism. We identified key differentially expressed genes (DEGs) between two groups with distinct prognosis. Dramatic correlation between TME hallmarks (angiogenesis and hypoxia) and metabolism pathways in malignant cells were illustrated by single-cell analysis. Our study lays a foundation for further research on the mechanism of tumor metabolism in colon cancer.

## 2. Materials and Methods

### 2.1. Data Acquisition

TCGA-COAD dataset including expression data, mutation data, and clinical information was obtained from The Cancer Genome Atlas (TCGA) database (https://portal.gdc.cancer.gov/). Expression profiles with fragments per kilobase million (FPKM) format were transformed into transcript per million (TPM) format. GSE17536 [18] and GSE161277 [19] datasets were downloaded from Gene Expression Omnibus (GEO) database (https://www.ncbi.nlm.nih.gov/geo/). Four metabolism pathways (glycolysis, PPP, FAO, and glutaminolysis) were obtained from Molecular Signatures Database [20] (MSigDB, https://www.gsea-msigdb.org/gsea/msigdb/).

### 2.2. Data Preprocessing

For TCGA-COAD dataset, samples without survival information were excluded, and survival time within 30 days to 10 years was restricted for all samples. 410 samples with expression data remained in TCGA-COAD dataset. For GSE17536 dataset, probes in chip files were converted to gene symbols. Samples were screened according to the same conditions as TCGA-COAD dataset. 170 samples with expression data remained in GSE17536 dataset.

### 2.3. Unsupervised Consensus Clustering

Firstly, single sample gene set enrichment analysis (ssGSEA) in GSVA R package was applied to calculate ssGSEA score of four metabolism pathways for each sample in TCGA-COAD dataset [21]. Then unsupervised consensus clustering was used to cluster samples based on the ssGSEA score of four metabolism pathways through ConsensusClusterPlus R package (v1.54.0) [22]. Kmdist algorithm and “Pearson” distance were used to conduct 500 bootstraps with each bootstrap containing at least 80% samples of TCGA-COAD dataset. Cluster number *k* was set from 2 to 10, and optimal *k* was determined according to cumulative distribution function (CDF) and area under CDF curve. The clustering was validated in GSE17536 dataset.

### 2.4. Mutation Analysis

Copy number variations (CNVs) and single nucleotide variations (SNVs) in TCGA-COAD dataset were analyzed separately. For CNV data, Genomic Identification of Significant Targets in Cancer (GISTIC) 2.0 software was employed to detect arm-level and focal CNVs based on hg38 reference genome [23]. The number of copies greater than 1 was considered the threshold of copy amplification and that less than −1 was considered the threshold of copy loss. R package of maftools was used to assess SNVs [24].

### 2.5. Tumor Microenvironment Analysis

To evaluate TME features in different clusters, several methodologies were applied. MCPcounter R package and the Proportion of Immune and Cancer cells (EPIC) methodology were used to calculate the enrichment score of immune cells [25, 26]. Estimation of STromal and Immune cells in MAlignant Tumors using Expression data (ESTIMATE) tool was introduced to evaluate immune infiltration and stromal infiltration by calculating immune score, stromal score, and ESTIMATE score based on signatures [27]. Gene sets of macrophage-related pathways including toll-like receptor signaling, natural killer cell mediated cytotoxicity, and antigen processing and presentation were obtained from MSigDB. Signatures of IFN-*γ* and cytotoxicity were obtained from previous research [28,29].

### 2.6. Differential Analysis between Clust12 and Clust3

DEGs between clust12 and clust3 were detected by limma R package under conditions of |log2 (fold change)| > log2 (1.5) and false discovery rate (FDR) < 0.05 [30]. String (https://cn.string-db.org/) online tool was applied to identify interactions among DEGs, and the interaction network was visualized by CytoScape (3.8.0) [31]. Degree of each DEG was analyzed by Analyze Network tool in CytoScape. Higher degree represents greater importance of the gene in the network. Annotation of gene ontology (GO) terms and Kyoto Encyclopedia of Genes and Genomes (KEGG) pathways was outputted by WebGestaltR package [32]. FDR < 0.05 was determined to screen significant GO terms and *P* < 0.05 was determined to screen significantly enriched KEGG pathways.

### 2.7. Identifying Immune Cells in scRNA-Seq Data

GSE161277 dataset containing scRNA-seq data was included for validating the reliability of molecular subtyping based on four metabolism pathways. Within the dataset, 13 samples, 4 patients, and 5 tissues (adenoma, blood, carcinoma, normal, and paracancer tissues) were included. Seurat R package was implemented to process scRNA-seq data [33]. Firstly, single cells were screened under conditions of each gene expressing at least in three cells and each cell expressing at least 250 genes. Then PercentageFeatureSet function was used to calculate the percentages of mitochondria and rRNA. Single cells with mitochondria <35% and unique molecular identifiers (UMI) > 100 within one cell were further screened. Log-normalization was conducted for normalizing single-cell data. FindVariableFeatures function was performed to identify highly variable genes based on “vst.” ScaleData function was conducted to scale the data and principle component analysis (PCA) was used to reduce the dimensionality. Then FindNeighbors and FindClusters functions were implemented to cluster cells when dim = 40 and resolution = 0.9. CD45 (PTPRC) was set as the marker to identify immune cells. Finally, 26188 immune cells remained.

### 2.8. Annotating Cell Types in Processed scRNA-seq Data

The expression data of 26188 immune cells was rescaled and highly variable genes were recalculated. Cells were clustered under dim = 35 and resolution = 0.3 by FindNeighbors and FindClusters functions in Seurat R package [33]. Then RunTSNE function was performed to reduce dimensionality for 26188 ells and identify cell subgroups with different samples, tissues, and patients. Classical immune markers were used to annotate cell subgroups. Differential genes were screened by using FindAllMarkers function with parameters of logfc = 0.5 and Minpct = 0.35 (*P* < 0.05). Only the top 5 differential genes were visualized. clusterProfiler *R* package was applied to annotate enriched KEGG pathways for differential genes [34].

### 2.9. Statistical Analysis

Statistical analysis was performed in R software (4.1.1). R packages and tools used in the study were indicated. Statistical methods were described in the corresponding sections. *P* < 0.05 was considered significant. “ns” indicates no significance. ^*∗*^*P* < 0.05, ^*∗∗*^*P* < 0.01, ^*∗∗∗*^*P* < 0.001, and ^*∗∗∗∗*^*P* < 0.0001.

## 3. Results

### 3.1. Constructing Molecular Subtypes Based on Metabolism-Related Pathways

**Figure 1:**
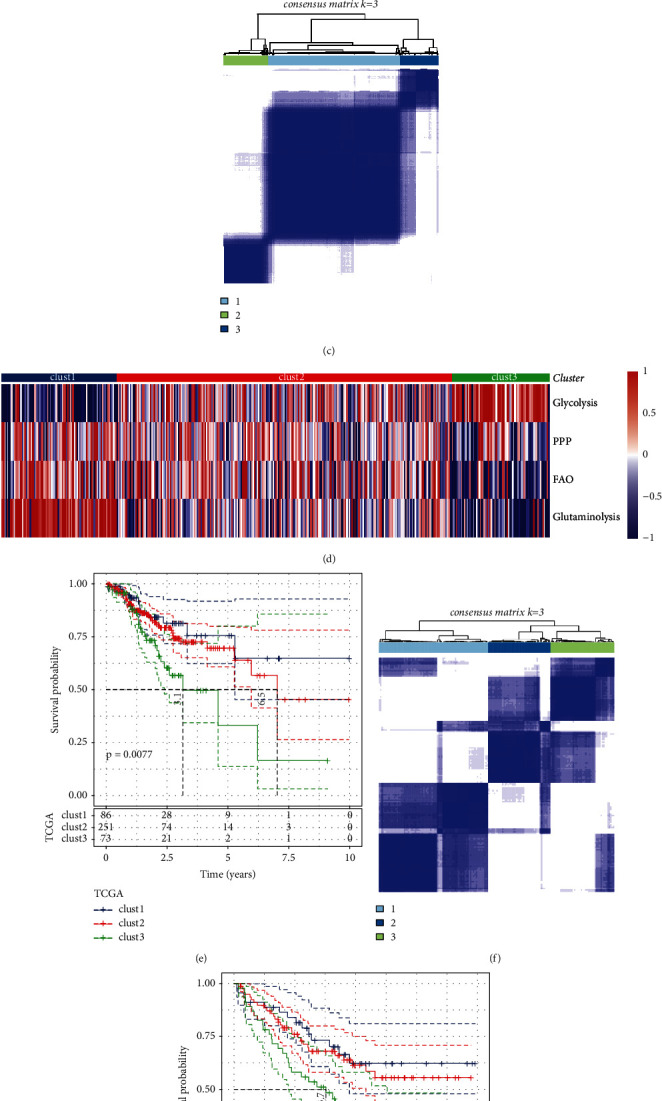
Constructing molecular subtypes based on metabolism pathways. ((a) and (b)) CDF curve and area under CDF curve when cluster number *k* = 2 to 10 analyzed by unsupervised consensus clustering. (c) Consensus matrix when *k* = 3 in TCGA-COAD dataset. (d) A heatmap of the enrichment of four metabolism pathways grouped by clusters. Blue and red indicate relatively low and high enrichment scores, respectively. (e) Kaplan-Meier survival curves of three clusters in TCGA-COAD dataset. (f) Consensus matrix when *k* = 3 in GSE17536 dataset. (g) Kaplan-Meier survival curves of three clusters in GSE17536 dataset. Log-rank test was conducted in (e) and (g).

The workflow of this study is shown in Supplementary Figure S1. For each sample in TCGA-COAD dataset, we calculated the enrichment score of four metabolism pathways, glycolysis, PPP, FAO, and glutaminolysis, by using ssGSEA (Supplementary Table S1). Then unsupervised consensus clustering was applied to cluster 410 samples based on the enrichment score of four metabolism pathways. Cluster number *k* was set from 2 to 10 in CDF analysis. Cluster number *k* = 3 was confirmed as optimal according to CDF and area under CDF curve (Figures 1(a) and 1(b)). Consensus matrix showed that samples were clearly divided into three clusters or molecular subtypes (Figure 1(c)). Three clusters showed different patterns of the enrichment of four metabolism pathways (Figure 1(d)). Glycolysis was the most enriched in clust3, while clust1 had the highest enrichment of glutaminolysis. The enrichment scores of PPP, FAO, and glutaminolysis were significantly higher in clust1 than in clust3. Over half of genes within four pathways (44 of 74) were differentially expressed among three clusters (Supplementary Figure S2).

Kaplan-Meier survival analysis revealed that three clusters had differential overall survival (*P*=0.0077, Figure 1(e)). Using the same methodology in GSE17536 dataset, 170 samples were divided into three clusters with distinct overall survival (*P*=0.0049, Figure 1(f)). Clust1 had the worst prognosis, while clust3 had the longest overall survival in both datasets (Figures 1(e) and 1(f)). PCA presented the different distribution of three clusters in two datasets (Supplementary Figure S3). Of the distribution of three clusters in different clinical features, we observed no significant difference in ages, genders, and stages (Supplementary Figure S4).

### 3.2. Differential Genomic Features among Three Subtypes

**Figure 2:**
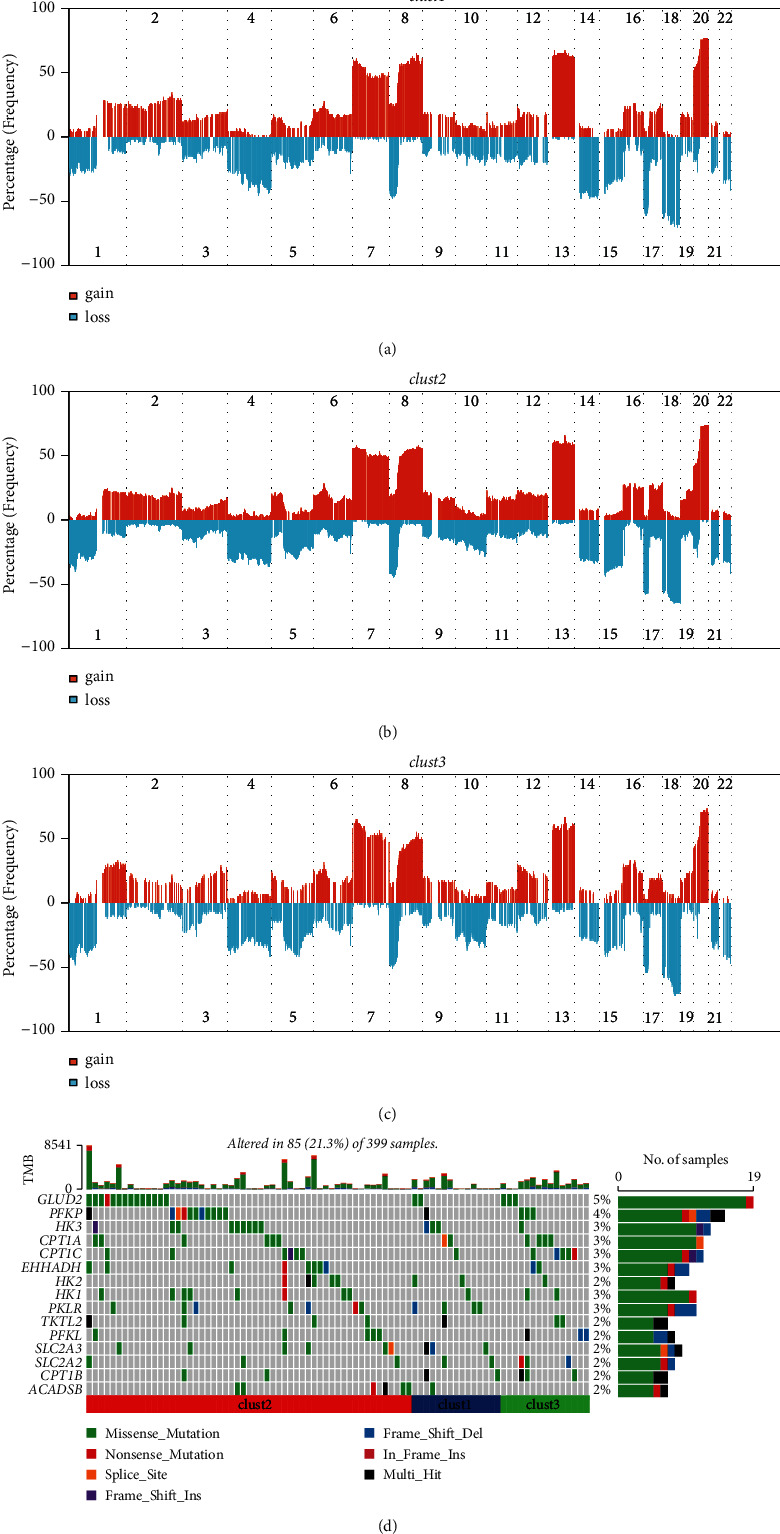
Genomic features of three clusters in TCGA-COAD dataset. ((a)–(c)) The proportion of CNVs in 22 chromosomes grouped by clust1, clust2, and clust3. (d) The top 10 mutated genes within metabolism pathways (Fisher's test, *P* <  0.05).

To know the genomic features of three subtypes, we applied gistic2 software to analyze the CNV data and visualized CNVs of 22 chromosomes (Figures 2(a)–2(c)). Three clusters showed obvious gain and loss of CNVs where gain of CNVs occurred largely in chromosomes 7, 8, 13, and 20, and loss of CNVs occurred largely in chromosome 18. Although similar CNV patterns were shown in three clusters, still a number of significantly differential CNVs were identified among them (Supplementary Figure S5). Clust2 had the most number of significantly amplified CNVs especially in chromosomes 17 and 20, compared to clust1 and clust3. Relatively lower counts of loss of CNVs were found in clust1 and clust2, but clust3 had more loss of CNVs than gain of CNVs itself (Supplementary Figure S5). In addition, we screened the top 15 mutated genes within four metabolism pathways based on SNV data (Figure 2(d)). The mutation frequencies ranged from 2% to 5%, and missense mutations contributed the majority of mutations. *GLUD2* was the top mutated gene, where missense mutations contributed the most of SNVs.

### 3.3. Differential TME among Three Clusters

**Figure 3:**
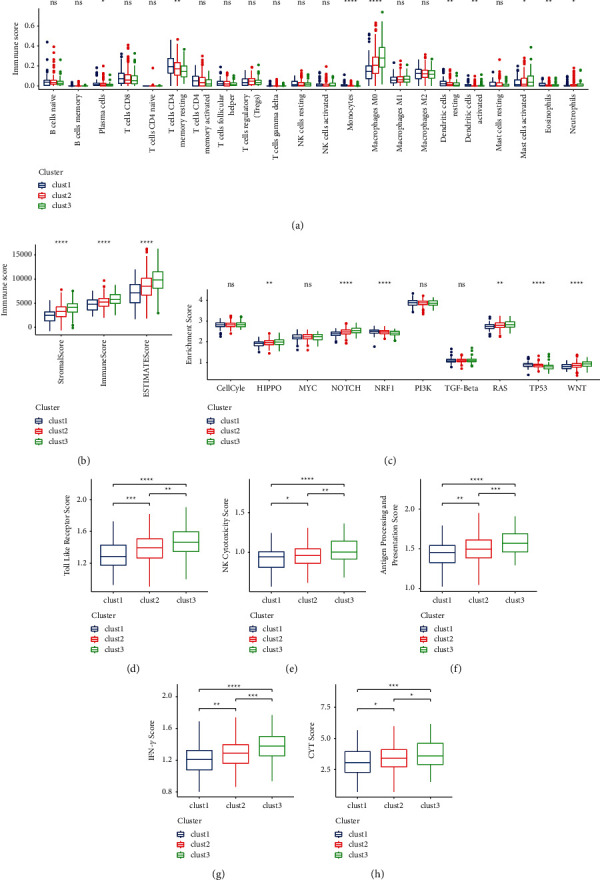
TME features of three clusters in TCGA-COAD dataset. (a) Estimated proportion of 22 immune cells. (b) Stromal score, immune score, and ESTIMATE score calculated by ESTIMATE. (c) Enrichment score of 10 oncogenic pathways. ((d)–(h)) Enrichment score of toll-like receptor, NK cytotoxicity, antigen processing and presentation, IFN-*γ*, and CYT. ANOVA was conducted. ns, no significance. ^*∗*^*P* <  0.05, ^*∗∗*^*P* <  0.01, ^*∗∗∗*^*P* < 0.001, and ^*∗∗∗∗*^*P* < 0.0001.

Next we used MCPcounter to evaluate the estimated proportion of 22 immune-related cells in three clusters in TCGA-COAD dataset. 9 of 22 immune-related cells showed a significant difference on the proportion among three clusters (Figure 3(a)). In particular, resting memory CD4 T cells and M0 macrophages consisted of a relatively high proportion among 22 immune-related cells. Clust1 had the highest proportion of resting memory CD4 T cells, while clust3 had the highest proportion of M0 macrophages (*P* < 0.01, Figure 3(a)). EPIC analysis on seven cell types showed that clust3 had significantly higher proportions of cancer-associated fibroblasts and endothelial cells (Supplementary Figure S6). Interestingly, clust1 with the longest overall survival had the least immune infiltration, and clust3 had the highest score of stromal and immune score (*P* < 0.0001, Figure 3(b)). We then assessed the enrichment of 10 oncogenic pathways [35] and observed that 6 of 10 oncogenic pathways were differentially enriched among three clusters (*P* < 0.01, Figure 3(c)). Hippo, Notch, RAS, and Wnt signaling pathways were more activated in clust3, while NRF1 and TP53 were more activated in clust1.

As M0 macrophages were identified to be significantly differentially enriched among three clusters, we further assessed the immune regulation related to macrophages. We selected three pathways related to macrophages from MSigDB including antigen processing and presentation, toll-like receptor signaling pathway, and natural killer (NK) cell mediated cytotoxicity. GSEA revealed that the three pathways were all most activated in clust3 (*P* < 0.01, Figures 3(d)–3(f)), which was accordant to the highest proportion of macrophages in clust3. In addition, clust3 also displayed high enrichment of interferon-*γ* (IFN-*γ*) and T cell cytotoxicity (CYT) (*P* < 0.05, Figures 3(g) and 3(h)). In GSE17536 dataset, similar results were presented (Supplementary Figure S7). Overall, clust3 showed more significantly active immune response than clust1 and clust2. Differential TME among three clusters indicated that metabolism pathways were possibly involved in complicated TME modulation.

### 3.4. Identifying Differentially Expressed Genes among Clusters

**Figure 4:**
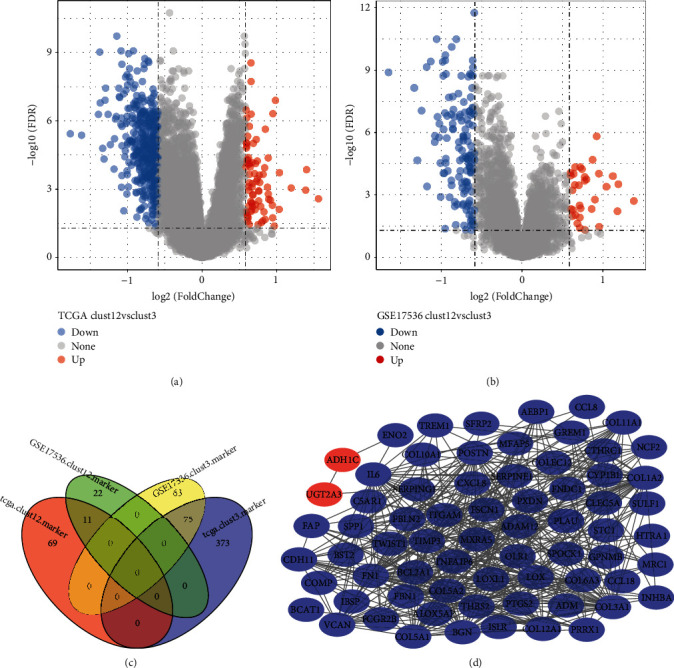
Identifying dysregulated genes related to prognosis. ((a) and (b)) DEGs between clust12 and clust3 in TCGA-COAD and GSE17536 datasets. Red and blue indicate upregulated and downregulated genes, respectively. (c) Venn plot of DEGs between clust12 and clust3 in two datasets. (d) Protein-protein interaction (PPI) analysis for 86 dysregulated genes. Blue indicates downregulated genes and red indicates upregulated genes.

Given that clust1 and clust2 had superior prognosis than clust3, we combined the data of clust1 and clust2 named clust12. By comparing expression profiles between clust12 and clust3 in TCGA-COAD and GSE17536 datasets, we identified a number of DEGs between two groups under conditions of |log2 fold change (FC)| > log2 (1.5) and FDR <0.05 (Figures 4(a) and 4(b)). 11 upregulated and 75 downregulated genes (clust12 versus clust3) were screened in both datasets (Figure 4(c)). Furthermore, based on 86 genes, we used String (https://cn.string-db.org/) to screen their interactions and found 69 interacted genes visualized by CytoScape (Figure 4(d)). Most of the genes were downregulated and only two genes (ADH1C and UGT2A3) within 69 interacted genes were upregulated. Genes with degree >10 were listed (Supplementary Table S2). Subsequently, we conducted GO and KEGG analysis on 69 genes. Within GO analysis, 92 terms of biological process, 20 terms of molecular function, and 13 terms of cellular component were annotated (Supplementary Table S3, FDR < 0.05). The top 10 enriched terms were visualized (Supplementary Figures S8(a)–S8(c)). In addition, 9 KEGG pathways were significantly enriched such as ECM-receptor interaction, focal adhesion, and PI3K-Akt signaling pathway (FDR < 0.05, Supplementary Figure S8(d)).

### 3.5. Identifying Eight Cell Types Based on scRNA-Seq Data

**Figure 5:**
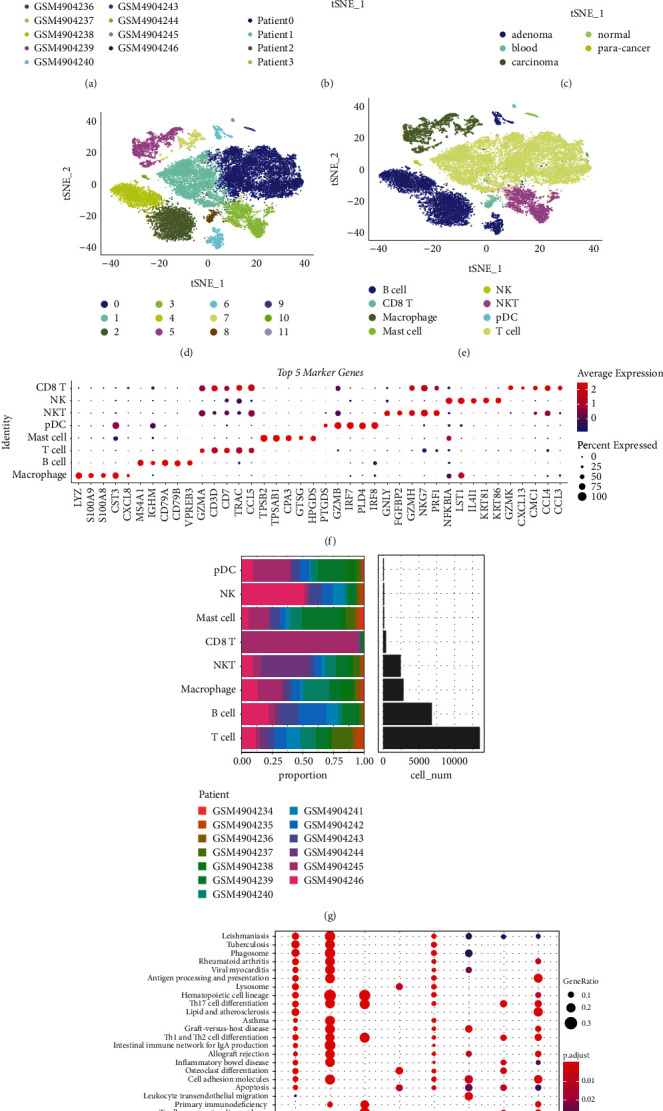
Single-cell analysis of GSE161277 dataset. ((a)–(c)) T-SNE plots of 26188 single cells grouped by samples (a), patients (b), and tissues (c). (d) Subgrouping of single cells. (e) Identification of eight cell types. (f) The top 5 DEGs (markers) of eight cell types. (g) The cell counts of different cell types and the distribution of samples. (h) Significantly enriched pathways of eight cell types.

To further verify the reliability of our molecular subtyping based on four metabolism pathways, we introduced scRNA-seq data of COAD and sought to distinguish malignant and nonmalignant cells. Single-cell data was preliminarily processed and screened to meet the standards of each gene expressed at least in three cells, each cell expressing at least 250 genes, each cell containing less than 35% mitochondria, and UMI >  100 for each cell (Supplementary Figures S9(a)–S9(c)). 45892 single cells remained. Then PCA was applied to diminish the dimensionality of the data. Cells were clustered into 34 clusters (resolution = 0.9) and CD45 marker was used to identify immune cells with a total number of 26188 (Supplementary Figures S9(d) and S9(e)). Subsequently, 26188 immune cells were further clustered under resolution = 0.3. 12 subgroups were identified, and t-SNE plots grouped by different samples, patients, and tissues were shown (Figures 5(a)–5(d)). By using immune markers from CellMarker (http://biocc.hrbmu.edu.cn/CellMarker/), 8 cell types were annotated, where T cells and B cells represented the majority (Figures 5(e)–5(g)). The top 5 DEGs among eight cell types were identified (Figure 5(f)), indicating the distinct expression features of them. KEGG analysis on 8 cell types manifested that 29 pathways were significantly enriched and many of them were related to immunity (Figure 5(h)).

### 3.6. Malignant Cells Were More Enriched in Clust3

**Figure 6:**
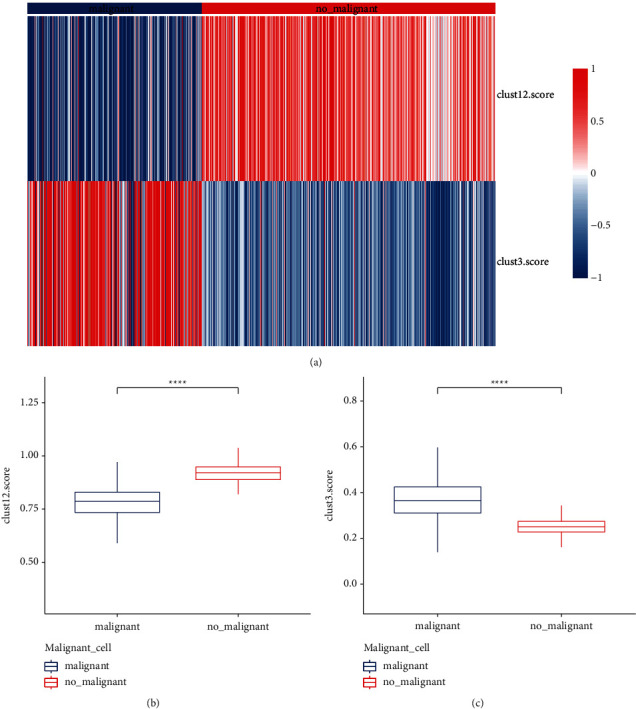
The distribution of clust12 score and clust3 score in malignant and nonmalignant cells. Wilcoxon test was performed. ^*∗∗∗∗*^*P* <  0.0001.

As the complicity of tumor tissues that may contain normal cells, we employed CopyKat to estimate genomic copy number profiles for more accurately distinguishing malignant and nonmalignant cells (Supplementary Figure S10) [36]. As a result, 8055 malignant cells and 18133 normal cells were distinguished. In the previous section, we identified 11 upregulated genes and 75 downregulated genes by comparing clust12 with clust3 (Figure 4(c)). We used ssGSEA to calculate their enrichment score of upregulated and downregulated genes in malignant and nonmalignant cells, respectively. Significantly differential enrichment of these genes was exhibited in two groups, with the 11 DEGs higher expression in nonmalignant cells and the 75 DEGs higher expression in malignant cells (*P* < 0.0001, Figure 6). The result demonstrated that the molecular subtyping based on metabolism pathways was reliable, and these dysregulated genes may be highly associated with the regulation of four metabolism pathways.

### 3.7. Metabolism of Malignant Cells Was Associated with TME

**Figure 7:**
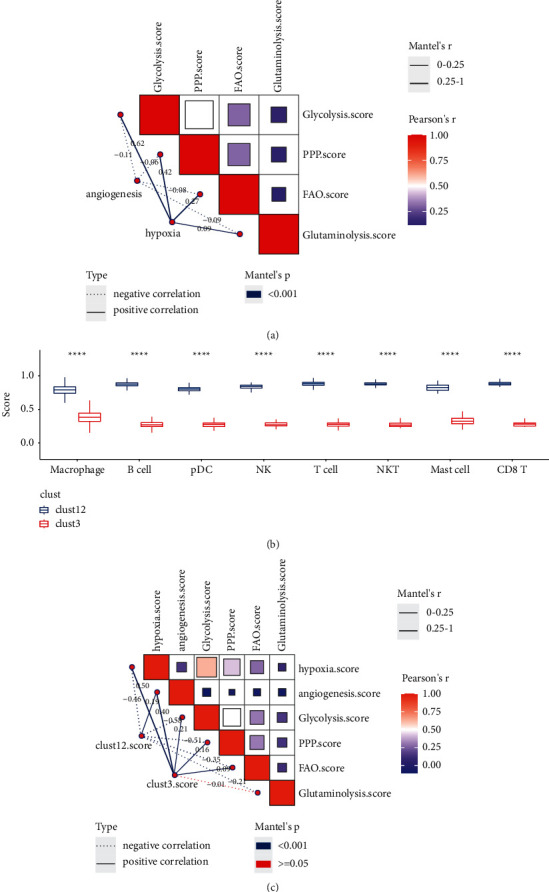
Analysis of the relation between TME and metabolism pathways. (a) Correlation analysis between hypoxia, angiogenesis, and four metabolism pathways. (b) Enrichment score of eight immune cell types in malignant and nonmalignant cells. (c) The correlation of clust12 score and clust3 score with hypoxia, angiogenesis, and four metabolism pathways. In (a) and (c), dashed lines indicate negative correlation and solid lines indicate positive correlation. Thicker lines indicate higher correlation. Pearson correlation analysis was conducted among pathways in top-right matrix. Mantel test was conducted between two matrixes. Wilcoxon test was performed in (b) and (c). ^*∗∗∗∗*^*P* < 0.0001.

To understand if TME made a difference on the metabolism of malignant cells, we introduced hypoxia and angiogenesis as indicators. Here hypoxia score was calculated by ssGSEA based on genes within hypoxia pathway, and angiogenesis score was calculated based on the signature from Masiero et al. [37]. Pearson correlation analysis revealed positive correlations between either two of the four metabolism pathways in malignant cells, especially between glycolysis and PPP (Figure 7(a)). In addition, we applied Mantel test to evaluate the relation of angiogenesis and hypoxia with four metabolism pathways. We observed that angiogenesis score was negatively correlated with four pathways, while positive correlation was shown between hypoxia and four pathways (Figure 7(a)). Notably, significantly high correlation coefficient was observed between hypoxia and glycolysis score (*R* = 0.62), indicating the strong interaction between two pathways.

Malignant cells mostly consisted of B cells, macrophages, and T cells (Supplementary Table S4). Notably, all detected cell types were more enriched in clust12 compared with clust3 (*P* < 0.0001, Figure 7(b)). Furthermore, we found that clust12 score was negatively correlated with hypoxia (*R* = −0.46), glycolysis (*R* = −0.58), PPP (*R* = −0.51), and FAO (*R* = −0.35) particularly (Figure 7(c)). Conversely, clust3 score was positively correlated with hypoxia and angiogenesis especially (*R* = 0.50 and 0.40, respectively, Figure 7(c)). In addition, we found that the enrichment score of hypoxia was significantly differential among three subtypes, where clust3 had the highest enrichment score (Supplementary Figure S11), which was consistent with the above result. Hypoxia and angiogenesis are important TME hallmarks in cancer. The results suggested that metabolism regulation of malignant cells was possibly regulated by hypoxia and angiogenesis, as well as TME. Additionally, the significantly differential distribution of immune cells between clust12 and clust3 proved that 86 markers (DEGs) were effective and reliable for distinguishing two groups in single-cell data.

## 4. Discussion

Tumor metabolism has a major impact in TME modulation [38,39], but the link between tumor metabolism and TME has not been clearly understood in COAD so far. In this study, we dug out key genes interacting with both four major metabolism pathways and TME by analyzing expression data and single-cell data. Initially, three clusters were established through unsupervised consensus clustering based on the enrichment score of four metabolism pathways. Given that clust1 and clust2 had similar overall survival, we combined the expression data of two clusters. Then 86 important DEGs were identified between clust12 and clust3, and 69 of 86 DEGs presented close interactions through PPI analysis.

We estimated that these DEGs may play a critical role in tumor metabolism and have a pronounced impact on COAD prognosis. As demonstrated in the single-cell analysis, malignant cells and nonmalignant cells had distinct expression levels of 86 DEGs (11 as clust12 markers and 75 as clust3 markers). Malignant cells had extremely high expression level of 75 DEGs associated with poor prognosis in clust3, which illustrated the important role of 75 DEGs in tumor progression, as well as the effectiveness and reasonability of subtyping based on four metabolism pathways.

Hypoxia and angiogenesis are two important characteristics in TME of solid tumor, which contributes to poor prognosis and drug resistance or inferior efficiency of immunotherapy [40–43]. Numerous studies have demonstrated that hypoxia can induce glycolytic flux through hypoxia-inducible factor-1*α* (HIF-1*α*). HIF-independent reprogramming is activated, which promotes expression of glucose transporters and enzymes involved in glucose pathway [44]. Immune cell metabolism can be altered HIF-1*α* activated glycolysis, where various immune cells are affected such as lymphocytes, dendritic cells, and macrophages [45,46]. Immunosuppressive cells including tumor-associated macrophages (TAMs) and myeloid-derived suppressor cells (MDSCs) are increased via HIF-1*α* signaling [47]. High activity of glycolysis also inhibits T cell function but favors infiltration of M2 macrophages which can suppress antitumor immune response and promote tumor growth [48]. In this study, striking correlation was observed between hypoxia and glycolysis (*R* = 0.62), between clust3 score and hypoxia (*R* = 0.50), between clust12 score and hypoxia (*R* = −0.46), and between clust12 score and glycolysis (*R* = −0.58). The correlation between clust3 score and glycolysis was relatively slight (*R* = 0.21), indicating that 75 markers were more involved in the signaling of hypoxia and thus leading to activated glycolysis in tumor tissues related to poor prognosis.

Besides glycolysis, PPP was also strongly correlated with hypoxia (*R* = 0.42), and clust12 score was shown to be negatively associated with PPP (*R* = −0.51). PPP is one of major pathway for glucose catabolism, and it is reprogrammed accompanied with the activation of glycolysis in tumor cells [49]. In the human colon cancer cell line (HT29), enzymes within PPP pathway are enhanced during cell cycle progression [50], suggesting that PPP is a potential target for inhibiting tumor cell proliferation [51]. High expression of 11 marker genes in clust12 may play an important role for alleviating PPP activation.

Angiogenesis is more activated in clust3 revealed from a correlation coefficient of 0.40 between clust3 score and angiogenesis. Several targeted drugs display antiangiogenesis effect in colon cancer, such as regorafenib [52], anti-interleukin-6 receptor antibody [53], and SARI [54]. Angiogenesis is regulated by innate immune cells such as TAMs, MDSCs, tumor-associated neutrophils (TANs), mast cells, and NK cells, which produce proangiogenic factors [55], and promote immunosuppressive TME [56]. Other two metabolism pathways, FAO and glutaminolysis, exhibited a relatively weak correlation with either hypoxia or angiogenesis, indicating that they may be less involved in dysregulated immunity in COAD.

## 5. Conclusions

In conclusion, this study depicted a crosstalk between tumor metabolism and TME features for COAD through integrated analysis of expression data and scRNA-seq data. Three molecular subtypes were constructed based on metabolism-related genes, and they performed significant differences in metabolism, genomic variations, immune infiltration, and enriched pathways, suggesting that the four metabolism pathways may regulate TME and thus affect COAD progression. Importantly, we identified 86 differentially expressed genes from three subtypes that were illustrated in scRNA-seq data to be highly associated with malignancy, hypoxia, and glycolysis. We envisioned that these key genes may provide new directions for further revealing the mechanism of metabolism in TME modulation.

## Data Availability

The datasets analyzed in this study can be found in [GSE17536] at https://www.ncbi.nlm.nih.gov/geo/query/acc.cgi?acc=GSE17536 and in [GSE161277] at https://www.ncbi.nlm.nih.gov/geo/query/acc.cgi?acc=GSE161277.

## Supplementary Materials

Supplementary Materials Supplementary Figure S1: the flowchart of this study. Supplementary Figure S2: expression of genes within four metabolism pathways in three clusters. Kruskal test was conducted. ns, no significance. ^*∗∗*^*P* <  0.01, ^*∗∗∗*^*P* < 0.001, and ^*∗∗∗∗*^*P* < 0.0001. Supplementary Figure S3: the distribution of three clusters in PCA plots. Supplementary Figure S4: the distribution of different clinical features in three clusters. *Chi*-square test was conducted. Supplementary Figure S5: significant CNVs detected by gistic2. Left line indicates chromosomes. Right line indicates significant CNVs (*q* value < 0.25). The bottom line indicates q values and the upper line indicates *G* score. Supplementary Figure S6: the enrichment score of immune cells in TCGA-COAD dataset analyzed by EPIC. Kruskal test was conducted. ns, no significant. ^*∗∗*^*P* <  0.01 and ^*∗∗∗∗*^*P* < 0.001. Supplementary Figure S7: TME features of three clusters in GSE17536 dataset. (a) Estimated proportion of 22 immune cells. (b) Stromal score, immune score, and ESTIMATE score calculated by ESTIMATE. (c) Enrichment score of 10 oncogenic pathways. ((d)–(h)) Enrichment scores of toll-like receptor, NK cytotoxicity, antigen processing and presentation, IFN-*γ*, and CYT. ANOVA was conducted. ns, no significance. ^*∗*^*P* < 0.05, ^*∗∗*^*P* < 0.01, ^*∗∗∗*^*P* < 0.01, and ^*∗∗∗∗*^*P* < 0.0001. Supplementary Figure S8: functional analysis on 69 genes within PPI network in TCGA-COAD dataset. ((a)–(c)) The top 10 significantly enriched terms in biological process (a), cellular component (b), and molecular function (c). (d) Nine significantly enriched KEGG pathways. Supplementary Figure S9: quality control and preprocessing of single-cell data. Supplementary Figure S10: the CNV heatmap of CopyKat for distinguishing malignant and nonmalignant cells. Orange indicates malignant cells and green indicates nonmalignant cells. Supplementary Figure S11: the ssGSEA enrichment score of hypoxia in three subtypes in TCGA-COAD dataset. Wilcoxon test was conducted. ^*∗∗∗∗*^*P* < 0.0001. Supplementary Table S1: a gene list of four metabolism pathways. Supplementary Table S2: screened genes with degree >10. Supplementary Table S3: the significantly enriched GO terms and KEGG pathways in TCGA-COAD dataset. Supplementary Table S4: the composed cell types of malignant cells.
