# Supplementary material for: Exploring the Interplay between Metabolism and Tumor Microenvironment Based on Four Major Metabolism Pathways in Colon Adenocarcinoma
Source: J Oncol. 2022 Jun 14;2022:2159794. doi: 10.1155/2022/2159794 (PMC9213191; doi:10.1155/2022/2159794)
Supplement: Supplementary Materials — Supplementary Figure S1: the flowchart of this study. Supplementary Figure S2: expression of genes within four metabolism pathways in three clusters. Kruskal test was conducted. ns, no significance. ∗∗P < 0.01, ∗∗∗P < 0.001, and ∗∗∗∗P < 0.0001. Supplementary Figure S3: the distribution of three clusters in PCA plots. Supplementary Figure S4: the distribution of different clinical features in three clusters. Chi-square test was conducted. Supplementary Figure S5: significant CNVs detected by gistic2. Left line indicates chromosomes. Right line indicates significant CNVs (q value < 0.25). The bottom line indicates q values and the upper line indicates G score. Supplementary Figure S6: the enrichment score of immune cells in TCGA-COAD dataset analyzed by EPIC. Kruskal test was conducted. ns, no significant. ∗∗P < 0.01 and ∗∗∗∗P < 0.001. Supplementary Figure S7: TME features of three clusters in GSE17536 dataset. (a) Estimated proportion of 22 immune cells. (b) Stromal score, immune score, and ESTIMATE score calculated by ESTIMATE. (c) Enrichment score of 10 oncogenic pathways. ((d)–(h)) Enrichment scores of toll-like receptor, NK cytotoxicity, antigen processing and presentation, IFN-γ, and CYT. ANOVA was conducted. ns, no significance. ∗P < 0.05, ∗∗P < 0.01, ∗∗∗P < 0.01, and ∗∗∗∗P < 0.0001. Supplementary Figure S8: functional analysis on 69 genes within PPI network in TCGA-COAD dataset. ((a)–(c)) The top 10 significantly enriched terms in biological process (a), cellular component (b), and molecular function (c). (d) Nine significantly enriched KEGG pathways. Supplementary Figure S9: quality control and preprocessing of single-cell data. Supplementary Figure S10: the CNV heatmap of CopyKat for distinguishing malignant and nonmalignant cells. Orange indicates malignant cells and green indicates nonmalignant cells. Supplementary Figure S11: the ssGSEA enrichment score of hypoxia in three subtypes in TCGA-COAD dataset. Wilcoxon test was conducted. ∗∗∗∗P < 0.0001. Supplem [file 2159794.f1.zip › 2159794.f1/Supplementary Table S1.pdf]

gene	pathway
HK1	Glycolysis
HK2	Glycolysis
HK3	Glycolysis
GCK	Glycolysis
GPI	Glycolysis
PFKM	Glycolysis
PFKP	Glycolysis
PFKL	Glycolysis
PFKFB1	Glycolysis
PFKFB2	Glycolysis
PFKFB3	Glycolysis
PFKFB4	Glycolysis
ALDOA	Glycolysis
ALDOB	Glycolysis
ALDOC	Glycolysis
TPI1	Glycolysis
GAPDH	Glycolysis
PGK1	Glycolysis
PGK2	Glycolysis
PGAM1	Glycolysis
PGAM2	Glycolysis
PGAM4	Glycolysis
BPGM	Glycolysis
ENO1	Glycolysis
ENO2	Glycolysis
ENO3	Glycolysis
PKLR	Glycolysis
PKM2	Glycolysis
LDHA	Glycolysis
LDHB	Glycolysis
LDHC	Glycolysis
SLC2A1	Glycolysis
SLC2A2	Glycolysis
SLC2A3	Glycolysis
SLC2A4	Glycolysis

SLC2A5	Glycolysis
G6PD	PPP
H6PD	PPP
PGLS	PPP
PGD	PPP
RPIA	PPP
RPE	PPP
RPEL1	PPP
TKT	PPP
TKTL1	PPP
TKTL2	PPP
TALDO1	PPP
EHHADH	PPP
ECI1	PPP
ECI2	PPP
HSD17B10	PPP
ACAA1	PPP
ACAA2	PPP
ACAT1	PPP
CPT1B	PPP
CPT1A	PPP
CPT1C	PPP
CPT2	PPP
ACADM	FAO
ACADVL	FAO
ACADS	FAO
ACADL	FAO
ACAD9	FAO
ACADSB	FAO
ACAD8	FAO
HADHA	FAO
HADHB	FAO
HADH	FAO
ECHS1	FAO
GLS	Glutaminolysis
GLS2	Glutaminolysis

GLUD1	Glutaminolysis
GLUD2	Glutaminolysis
GPT	Glutaminolysis
GOT1	Glutaminolysis
GOT2	Glutaminolysis
