# Supplementary material for: Exploring the Interplay between Metabolism and Tumor Microenvironment Based on Four Major Metabolism Pathways in Colon Adenocarcinoma
Source: J Oncol. 2022 Jun 14;2022:2159794. doi: 10.1155/2022/2159794 (PMC9213191; doi:10.1155/2022/2159794)
Supplement: Supplementary Materials — Supplementary Figure S1: the flowchart of this study. Supplementary Figure S2: expression of genes within four metabolism pathways in three clusters. Kruskal test was conducted. ns, no significance. ∗∗P < 0.01, ∗∗∗P < 0.001, and ∗∗∗∗P < 0.0001. Supplementary Figure S3: the distribution of three clusters in PCA plots. Supplementary Figure S4: the distribution of different clinical features in three clusters. Chi-square test was conducted. Supplementary Figure S5: significant CNVs detected by gistic2. Left line indicates chromosomes. Right line indicates significant CNVs (q value < 0.25). The bottom line indicates q values and the upper line indicates G score. Supplementary Figure S6: the enrichment score of immune cells in TCGA-COAD dataset analyzed by EPIC. Kruskal test was conducted. ns, no significant. ∗∗P < 0.01 and ∗∗∗∗P < 0.001. Supplementary Figure S7: TME features of three clusters in GSE17536 dataset. (a) Estimated proportion of 22 immune cells. (b) Stromal score, immune score, and ESTIMATE score calculated by ESTIMATE. (c) Enrichment score of 10 oncogenic pathways. ((d)–(h)) Enrichment scores of toll-like receptor, NK cytotoxicity, antigen processing and presentation, IFN-γ, and CYT. ANOVA was conducted. ns, no significance. ∗P < 0.05, ∗∗P < 0.01, ∗∗∗P < 0.01, and ∗∗∗∗P < 0.0001. Supplementary Figure S8: functional analysis on 69 genes within PPI network in TCGA-COAD dataset. ((a)–(c)) The top 10 significantly enriched terms in biological process (a), cellular component (b), and molecular function (c). (d) Nine significantly enriched KEGG pathways. Supplementary Figure S9: quality control and preprocessing of single-cell data. Supplementary Figure S10: the CNV heatmap of CopyKat for distinguishing malignant and nonmalignant cells. Orange indicates malignant cells and green indicates nonmalignant cells. Supplementary Figure S11: the ssGSEA enrichment score of hypoxia in three subtypes in TCGA-COAD dataset. Wilcoxon test was conducted. ∗∗∗∗P < 0.0001. Supplem [file 2159794.f1.zip › 2159794.f1/Supplementary Table S3.pdf]

geneSet	description	enrichmentRatio	pValue	FDR	DB
hsa04974	Protein digestion and absorption	16.12	0.0000	0.0001	pathway_KEGG
hsa04512	ECM-receptor interaction	12.94	0.0000	0.0001	pathway_KEGG
hsa04145	Phagosome	9.14	0.0000	0.0002	pathway_KEGG
hsa04610	Complement and coagulation cascades	9.47	0.0002	0.0118	pathway_KEGG
hsa04510	Focal adhesion	5.52	0.0002	0.0128	pathway_KEGG
hsa05146	Amoebiasis	8.14	0.0003	0.0161	pathway_KEGG
hsa04933	AGE-RAGE signaling pathway in diabetic complications	7.72	0.0004	0.0177	pathway_KEGG
hsa05165	Human papillomavirus infection	3.97	0.0007	0.0255	pathway_KEGG
hsa04151	PI3K-Akt signaling pathway	3.60	0.0013	0.0431	pathway_KEGG
GO:0043062	extracellular structure organization	15.02	0.0000	0.0000	geneontology_Biological_Process
GO:0030198	extracellular matrix organization	17.22	0.0000	0.0000	geneontology_Biological_Process
GO:0030199	collagen fibril organization	47.75	0.0000	0.0000	geneontology_Biological_Process
GO:0048514	blood vessel morphogenesis	7.15	0.0000	0.0000	geneontology_Biological_Process
GO:0001503	ossification	8.45	0.0000	0.0000	geneontology_Biological_Process
GO:0001525	angiogenesis	6.67	0.0000	0.0000	geneontology_Biological_Process
GO:0045765	regulation of angiogenesis	9.09	0.0000	0.0000	geneontology_Biological_Process
GO:0001501	skeletal system development	6.68	0.0000	0.0000	geneontology_Biological_Process
GO:1901342	regulation of vasculature development	8.15	0.0000	0.0000	geneontology_Biological_Process
GO:0045766	positive regulation of angiogenesis	11.87	0.0000	0.0000	geneontology_Biological_Process
GO:1904018	positive regulation of vasculature development	10.68	0.0000	0.0001	geneontology_Biological_Process
GO:0001649	osteoblast differentiation	9.48	0.0000	0.0002	geneontology_Biological_Process
GO:0010469	regulation of signaling receptor activity	5.56	0.0000	0.0002	geneontology_Biological_Process
GO:0031214	biomineral tissue development	11.98	0.0000	0.0007	geneontology_Biological_Process
GO:0035987	endodermal cell differentiation	22.82	0.0000	0.0009	geneontology_Biological_Process
GO:0071674	mononuclear cell migration	15.04	0.0000	0.0009	geneontology_Biological_Process
GO:0050900	leukocyte migration	5.48	0.0000	0.0012	geneontology_Biological_Process
GO:0001706	endoderm formation	20.70	0.0000	0.0012	geneontology_Biological_Process
GO:0030282	bone mineralization	13.87	0.0000	0.0012	geneontology_Biological_Process
GO:1900047	negative regulation of hemostasis	18.16	0.0000	0.0021	geneontology_Biological_Process
GO:0032102	negative regulation of response to external stimulus	6.31	0.0000	0.0027	geneontology_Biological_Process
GO:0002526	acute inflammatory response	9.09	0.0000	0.0027	geneontology_Biological_Process
GO:0002548	monocyte chemotaxis	16.79	0.0000	0.0027	geneontology_Biological_Process
GO:0010573	vascular endothelial growth factor production	27.38	0.0000	0.0028	geneontology_Biological_Process
GO:0010574	regulation of vascular endothelial growth factor production	27.38	0.0000	0.0028	geneontology_Biological_Process
GO:0001704	formation of primary germ layer	11.24	0.0000	0.0029	geneontology_Biological_Process
GO:0042730	fibrinolysis	26.37	0.0000	0.0029	geneontology_Biological_Process
GO:0033627	cell adhesion mediated by integrin	15.89	0.0000	0.0029	geneontology_Biological_Process
GO:0042476	odontogenesis	10.79	0.0000	0.0035	geneontology_Biological_Process
GO:0048598	embryonic morphogenesis	4.59	0.0000	0.0039	geneontology_Biological_Process
GO:0002063	chondrocyte development	23.73	0.0000	0.0040	geneontology_Biological_Process
GO:0007492	endoderm development	14.13	0.0000	0.0045	geneontology_Biological_Process
GO:0042060	wound healing	4.41	0.0000	0.0051	geneontology_Biological_Process
GO:0030514	negative regulation of BMP signaling pathway	20.94	0.0000	0.0060	geneontology_Biological_Process
GO:1900046	regulation of hemostasis	13.09	0.0000	0.0060	geneontology_Biological_Process
GO:0050727	regulation of inflammatory response	5.30	0.0000	0.0066	geneontology_Biological_Process
GO:0060840	artery development	12.36	0.0000	0.0075	geneontology_Biological_Process
GO:0010951	negative regulation of endopeptidase activity	7.08	0.0001	0.0080	geneontology_Biological_Process
GO:0048705	skeletal system morphogenesis	7.04	0.0001	0.0080	geneontology_Biological_Process
GO:1903035	negative regulation of response to wounding	11.87	0.0001	0.0085	geneontology_Biological_Process
GO:0010466	negative regulation of peptidase activity	6.92	0.0001	0.0085	geneontology_Biological_Process
GO:0033628	regulation of cell adhesion mediated by integrin	18.25	0.0001	0.0086	geneontology_Biological_Process
GO:2000147	positive regulation of cell motility	4.41	0.0001	0.0095	geneontology_Biological_Process
GO:0002673	regulation of acute inflammatory response	10.99	0.0001	0.0109	geneontology_Biological_Process
GO:0051272	positive regulation of cellular component movement	4.31	0.0001	0.0109	geneontology_Biological_Process
GO:0007178	transmembrane receptor protein serine/threonine kinase signaling	5.43	0.0001	0.0121	geneontology_Biological_Process
GO:0061448	connective tissue development	6.39	0.0001	0.0123	geneontology_Biological_Process
GO:0030728	ovulation	31.41	0.0001	0.0128	geneontology_Biological_Process
GO:0002062	chondrocyte differentiation	10.35	0.0001	0.0133	geneontology_Biological_Process
GO:0040017	positive regulation of locomotion	4.14	0.0001	0.0138	geneontology_Biological_Process
GO:0030195	negative regulation of blood coagulation	14.83	0.0001	0.0161	geneontology_Biological_Process
GO:0045992	negative regulation of embryonic development	28.10	0.0002	0.0164	geneontology_Biological_Process
GO:0007369	gastrulation	7.36	0.0002	0.0164	geneontology_Biological_Process
GO:0050819	negative regulation of coagulation	14.53	0.0002	0.0165	geneontology_Biological_Process
GO:0002237	response to molecule of bacterial origin	5.03	0.0002	0.0172	geneontology_Biological_Process
GO:0060348	bone development	7.22	0.0002	0.0173	geneontology_Biological_Process
GO:0007596	blood coagulation	4.98	0.0002	0.0179	geneontology_Biological_Process
GO:0050817	coagulation	4.93	0.0002	0.0189	geneontology_Biological_Process
GO:0051216	cartilage development	7.03	0.0002	0.0190	geneontology_Biological_Process
GO:0007599	hemostasis	4.89	0.0002	0.0191	geneontology_Biological_Process
GO:0010575	positive regulation of vascular endothelial growth factor producti	25.43	0.0002	0.0193	geneontology_Biological_Process
GO:0097529	myeloid leukocyte migration	6.59	0.0003	0.0255	geneontology_Biological_Process

GO:0034103	regulation of tissue remodeling	12.27	0.0003	0.0272	geneontology_Biological_Process
GO:0030335	positive regulation of cell migration	4.08	0.0003	0.0274	geneontology_Biological_Process
GO:0010810	regulation of cell-substrate adhesion	6.43	0.0003	0.0278	geneontology_Biological_Process
GO:0030509	BMP signaling pathway	7.95	0.0004	0.0341	geneontology_Biological_Process
GO:0030510	regulation of BMP signaling pathway	11.30	0.0004	0.0352	geneontology_Biological_Process
GO:0030595	leukocyte chemotaxis	6.10	0.0004	0.0352	geneontology_Biological_Process
GO:0050729	positive regulation of inflammatory response	7.74	0.0004	0.0368	geneontology_Biological_Process
GO:0002683	negative regulation of immune system process	4.31	0.0005	0.0383	geneontology_Biological_Process
GO:0052547	regulation of peptidase activity	4.29	0.0005	0.0387	geneontology_Biological_Process
GO:0070664	negative regulation of leukocyte proliferation	10.79	0.0005	0.0387	geneontology_Biological_Process
GO:1903053	regulation of extracellular matrix organization	19.07	0.0005	0.0387	geneontology_Biological_Process
GO:0050878	regulation of body fluid levels	3.80	0.0005	0.0393	geneontology_Biological_Process
GO:0030193	regulation of blood coagulation	10.63	0.0005	0.0393	geneontology_Biological_Process
GO:0061045	negative regulation of wound healing	10.63	0.0005	0.0393	geneontology_Biological_Process
GO:0071772	response to BMP	7.42	0.0005	0.0396	geneontology_Biological_Process
GO:0071773	cellular response to BMP stimulus	7.42	0.0005	0.0396	geneontology_Biological_Process
GO:0018149	peptide cross-linking	18.41	0.0006	0.0397	geneontology_Biological_Process
GO:0045861	negative regulation of proteolysis	4.81	0.0006	0.0402	geneontology_Biological_Process
GO:0007568	aging	4.81	0.0006	0.0402	geneontology_Biological_Process
GO:0090596	sensory organ morphogenesis	5.74	0.0006	0.0402	geneontology_Biological_Process
GO:0050818	regulation of coagulation	10.32	0.0006	0.0402	geneontology_Biological_Process
GO:0007507	heart development	3.72	0.0006	0.0408	geneontology_Biological_Process
GO:0010470	regulation of gastrulation	17.80	0.0006	0.0408	geneontology_Biological_Process
GO:0150063	visual system development	4.68	0.0007	0.0447	geneontology_Biological_Process
GO:0048880	sensory system development	4.63	0.0007	0.0466	geneontology_Biological_Process
GO:0048736	appendage development	6.95	0.0007	0.0466	geneontology_Biological_Process
GO:0060173	limb development	6.95	0.0007	0.0466	geneontology_Biological_Process
GO:1903034	regulation of response to wounding	6.90	0.0008	0.0477	geneontology_Biological_Process
GO:0031589	cell-substrate adhesion	4.58	0.0008	0.0482	geneontology_Biological_Process
GO:0032496	response to lipopolysaccharide	4.56	0.0008	0.0487	geneontology_Biological_Process
GO:0031012	extracellular matrix	17.20	0.0000	0.0000	geneontology_Cellular_Component
GO:0062023	collagen-containing extracellular matrix	18.92	0.0000	0.0000	geneontology_Cellular_Component
GO:0005581	collagen trimer	34.01	0.0000	0.0000	geneontology_Cellular_Component
GO:0005788	endoplasmic reticulum lumen	12.17	0.0000	0.0000	geneontology_Cellular_Component
GO:0005583	fibrillar collagen trimer	109.57	0.0000	0.0000	geneontology_Cellular_Component
GO:0098643	banded collagen fibril	109.57	0.0000	0.0000	geneontology_Cellular_Component
GO:0044420	extracellular matrix component	35.68	0.0000	0.0000	geneontology_Cellular_Component
GO:0098644	complex of collagen trimers	60.87	0.0000	0.0000	geneontology_Cellular_Component
GO:0005604	basement membrane	18.01	0.0000	0.0001	geneontology_Cellular_Component
GO:0070821	tertiary granule membrane	17.19	0.0001	0.0057	geneontology_Cellular_Component
GO:0035579	specific granule membrane	14.61	0.0002	0.0099	geneontology_Cellular_Component
GO:0070820	tertiary granule	9.21	0.0002	0.0116	geneontology_Cellular_Component
GO:0031091	platelet alpha granule	11.24	0.0004	0.0231	geneontology_Cellular_Component
GO:0005201	extracellular matrix structural constituent	26.24	0.0000	0.0000	geneontology_Molecular_Function
GO:0030020	extracellular matrix structural constituent conferring tensile streng	43.22	0.0000	0.0000	geneontology_Molecular_Function
GO:0005539	glycosaminoglycan binding	12.79	0.0000	0.0000	geneontology_Molecular_Function
GO:0005178	integrin binding	14.39	0.0000	0.0000	geneontology_Molecular_Function
GO:0008201	heparin binding	12.14	0.0000	0.0000	geneontology_Molecular_Function
GO:0002020	protease binding	10.90	0.0000	0.0005	geneontology_Molecular_Function
GO:1901681	sulfur compound binding	8.53	0.0000	0.0005	geneontology_Molecular_Function
GO:0050840	extracellular matrix binding	19.90	0.0000	0.0006	geneontology_Molecular_Function
GO:0050839	cell adhesion molecule binding	4.65	0.0000	0.0017	geneontology_Molecular_Function
GO:0061134	peptidase regulator activity	8.30	0.0000	0.0017	geneontology_Molecular_Function
GO:0048018	receptor ligand activity	5.13	0.0000	0.0017	geneontology_Molecular_Function
GO:0043394	proteoglycan binding	23.88	0.0000	0.0017	geneontology_Molecular_Function
GO:0048407	platelet-derived growth factor binding	48.84	0.0000	0.0019	geneontology_Molecular_Function
GO:0030545	receptor regulator activity	4.83	0.0000	0.0023	geneontology_Molecular_Function
GO:0061135	endopeptidase regulator activity	8.74	0.0001	0.0038	geneontology_Molecular_Function
GO:0004867	serine-type endopeptidase inhibitor activity	13.02	0.0002	0.0135	geneontology_Molecular_Function
GO:0005518	collagen binding	13.02	0.0002	0.0135	geneontology_Molecular_Function
GO:0005125	cytokine activity	6.51	0.0003	0.0158	geneontology_Molecular_Function
GO:0004866	endopeptidase inhibitor activity	7.65	0.0005	0.0236	geneontology_Molecular_Function
GO:0030414	peptidase inhibitor activity	7.52	0.0005	0.0243	geneontology_Molecular_Function
