# Supplementary figures and images for: Exploring the Interplay between Metabolism and Tumor Microenvironment Based on Four Major Metabolism Pathways in Colon Adenocarcinoma
Source: J Oncol. 2022 Jun 14;2022:2159794. doi: 10.1155/2022/2159794 (PMC9213191; doi:10.1155/2022/2159794)

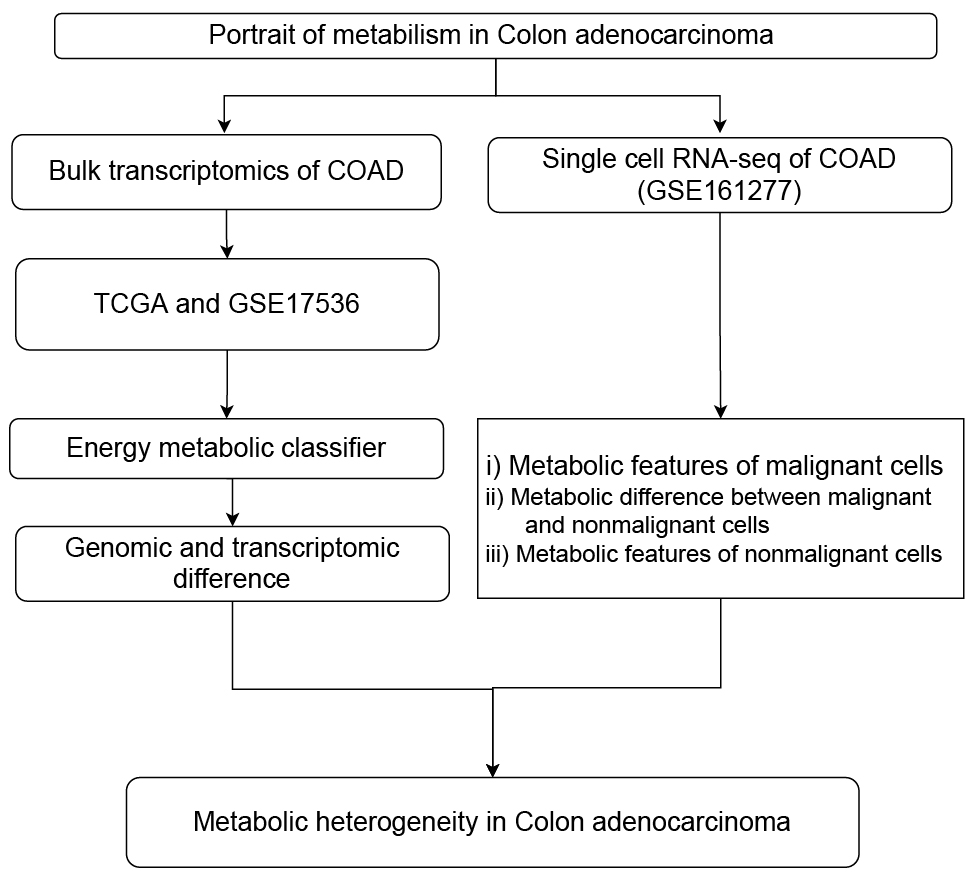

Supplement: Supplementary Materials — Supplementary Figure S1: the flowchart of this study. Supplementary Figure S2: expression of genes within four metabolism pathways in three clusters. Kruskal test was conducted. ns, no significance. ∗∗P < 0.01, ∗∗∗P < 0.001, and ∗∗∗∗P < 0.0001. Supplementary Figure S3: the distribution of three clusters in PCA plots. Supplementary Figure S4: the distribution of different clinical features in three clusters. Chi-square test was conducted. Supplementary Figure S5: significant CNVs detected by gistic2. Left line indicates chromosomes. Right line indicates significant CNVs (q value < 0.25). The bottom line indicates q values and the upper line indicates G score. Supplementary Figure S6: the enrichment score of immune cells in TCGA-COAD dataset analyzed by EPIC. Kruskal test was conducted. ns, no significant. ∗∗P < 0.01 and ∗∗∗∗P < 0.001. Supplementary Figure S7: TME features of three clusters in GSE17536 dataset. (a) Estimated proportion of 22 immune cells. (b) Stromal score, immune score, and ESTIMATE score calculated by ESTIMATE. (c) Enrichment score of 10 oncogenic pathways. ((d)–(h)) Enrichment scores of toll-like receptor, NK cytotoxicity, antigen processing and presentation, IFN-γ, and CYT. ANOVA was conducted. ns, no significance. ∗P < 0.05, ∗∗P < 0.01, ∗∗∗P < 0.01, and ∗∗∗∗P < 0.0001. Supplementary Figure S8: functional analysis on 69 genes within PPI network in TCGA-COAD dataset. ((a)–(c)) The top 10 significantly enriched terms in biological process (a), cellular component (b), and molecular function (c). (d) Nine significantly enriched KEGG pathways. Supplementary Figure S9: quality control and preprocessing of single-cell data. Supplementary Figure S10: the CNV heatmap of CopyKat for distinguishing malignant and nonmalignant cells. Orange indicates malignant cells and green indicates nonmalignant cells. Supplementary Figure S11: the ssGSEA enrichment score of hypoxia in three subtypes in TCGA-COAD dataset. Wilcoxon test was conducted. ∗∗∗∗P < 0.0001. Supplem [file 2159794.f1.zip › 2159794.f1/Supplementary Figure S1.jpg]

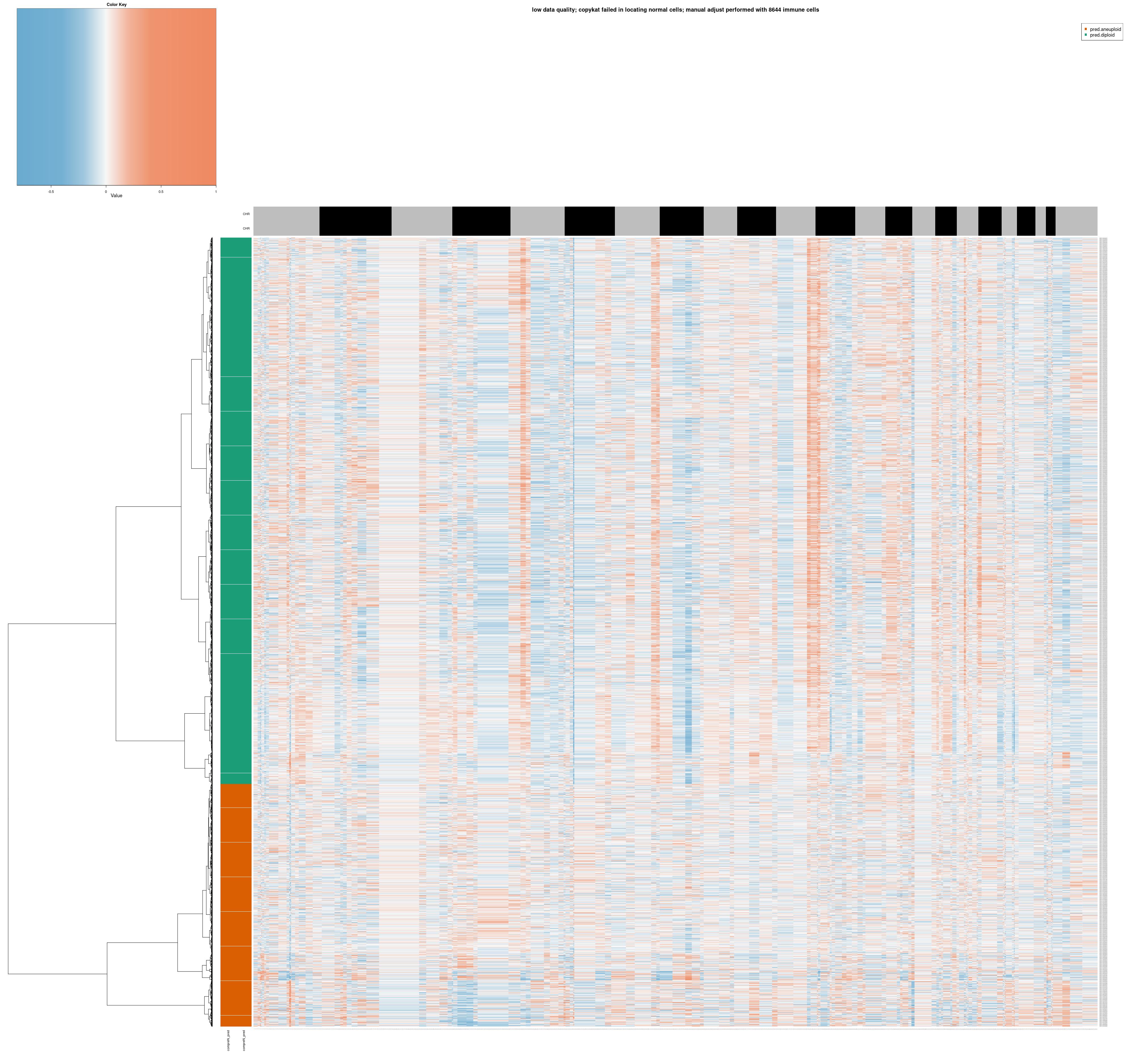

Supplement: Supplementary Materials — Supplementary Figure S1: the flowchart of this study. Supplementary Figure S2: expression of genes within four metabolism pathways in three clusters. Kruskal test was conducted. ns, no significance. ∗∗P < 0.01, ∗∗∗P < 0.001, and ∗∗∗∗P < 0.0001. Supplementary Figure S3: the distribution of three clusters in PCA plots. Supplementary Figure S4: the distribution of different clinical features in three clusters. Chi-square test was conducted. Supplementary Figure S5: significant CNVs detected by gistic2. Left line indicates chromosomes. Right line indicates significant CNVs (q value < 0.25). The bottom line indicates q values and the upper line indicates G score. Supplementary Figure S6: the enrichment score of immune cells in TCGA-COAD dataset analyzed by EPIC. Kruskal test was conducted. ns, no significant. ∗∗P < 0.01 and ∗∗∗∗P < 0.001. Supplementary Figure S7: TME features of three clusters in GSE17536 dataset. (a) Estimated proportion of 22 immune cells. (b) Stromal score, immune score, and ESTIMATE score calculated by ESTIMATE. (c) Enrichment score of 10 oncogenic pathways. ((d)–(h)) Enrichment scores of toll-like receptor, NK cytotoxicity, antigen processing and presentation, IFN-γ, and CYT. ANOVA was conducted. ns, no significance. ∗P < 0.05, ∗∗P < 0.01, ∗∗∗P < 0.01, and ∗∗∗∗P < 0.0001. Supplementary Figure S8: functional analysis on 69 genes within PPI network in TCGA-COAD dataset. ((a)–(c)) The top 10 significantly enriched terms in biological process (a), cellular component (b), and molecular function (c). (d) Nine significantly enriched KEGG pathways. Supplementary Figure S9: quality control and preprocessing of single-cell data. Supplementary Figure S10: the CNV heatmap of CopyKat for distinguishing malignant and nonmalignant cells. Orange indicates malignant cells and green indicates nonmalignant cells. Supplementary Figure S11: the ssGSEA enrichment score of hypoxia in three subtypes in TCGA-COAD dataset. Wilcoxon test was conducted. ∗∗∗∗P < 0.0001. Supplem [file 2159794.f1.zip › 2159794.f1/Supplementary Figure S10.jpeg]

Cluster 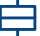 clust3 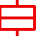 clust2 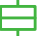 clust1

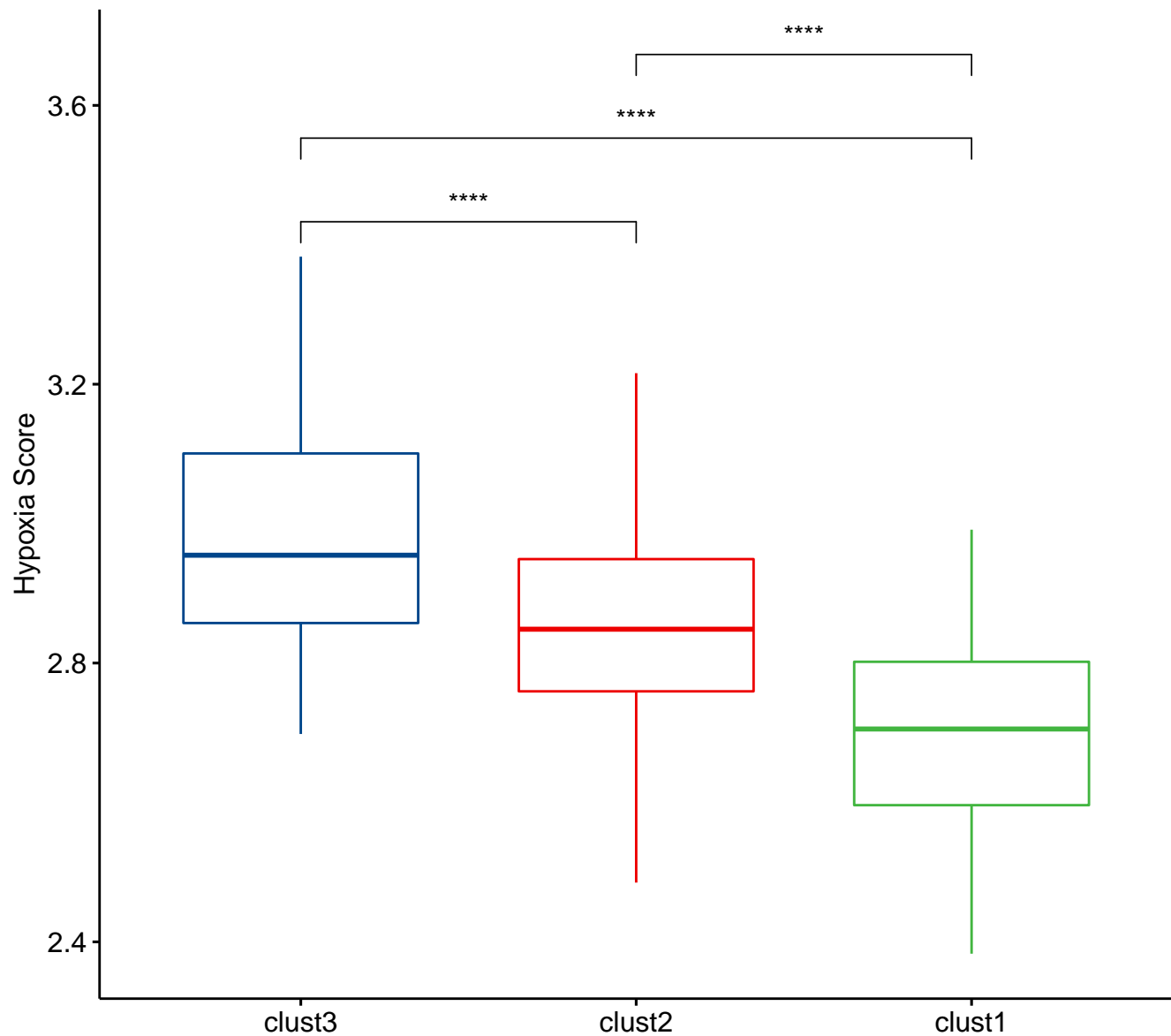

Supplement: Supplementary Materials — Supplementary Figure S1: the flowchart of this study. Supplementary Figure S2: expression of genes within four metabolism pathways in three clusters. Kruskal test was conducted. ns, no significance. ∗∗P < 0.01, ∗∗∗P < 0.001, and ∗∗∗∗P < 0.0001. Supplementary Figure S3: the distribution of three clusters in PCA plots. Supplementary Figure S4: the distribution of different clinical features in three clusters. Chi-square test was conducted. Supplementary Figure S5: significant CNVs detected by gistic2. Left line indicates chromosomes. Right line indicates significant CNVs (q value < 0.25). The bottom line indicates q values and the upper line indicates G score. Supplementary Figure S6: the enrichment score of immune cells in TCGA-COAD dataset analyzed by EPIC. Kruskal test was conducted. ns, no significant. ∗∗P < 0.01 and ∗∗∗∗P < 0.001. Supplementary Figure S7: TME features of three clusters in GSE17536 dataset. (a) Estimated proportion of 22 immune cells. (b) Stromal score, immune score, and ESTIMATE score calculated by ESTIMATE. (c) Enrichment score of 10 oncogenic pathways. ((d)–(h)) Enrichment scores of toll-like receptor, NK cytotoxicity, antigen processing and presentation, IFN-γ, and CYT. ANOVA was conducted. ns, no significance. ∗P < 0.05, ∗∗P < 0.01, ∗∗∗P < 0.01, and ∗∗∗∗P < 0.0001. Supplementary Figure S8: functional analysis on 69 genes within PPI network in TCGA-COAD dataset. ((a)–(c)) The top 10 significantly enriched terms in biological process (a), cellular component (b), and molecular function (c). (d) Nine significantly enriched KEGG pathways. Supplementary Figure S9: quality control and preprocessing of single-cell data. Supplementary Figure S10: the CNV heatmap of CopyKat for distinguishing malignant and nonmalignant cells. Orange indicates malignant cells and green indicates nonmalignant cells. Supplementary Figure S11: the ssGSEA enrichment score of hypoxia in three subtypes in TCGA-COAD dataset. Wilcoxon test was conducted. ∗∗∗∗P < 0.0001. Supplem [file 2159794.f1.zip › 2159794.f1/Supplementary Figure S11.pdf]

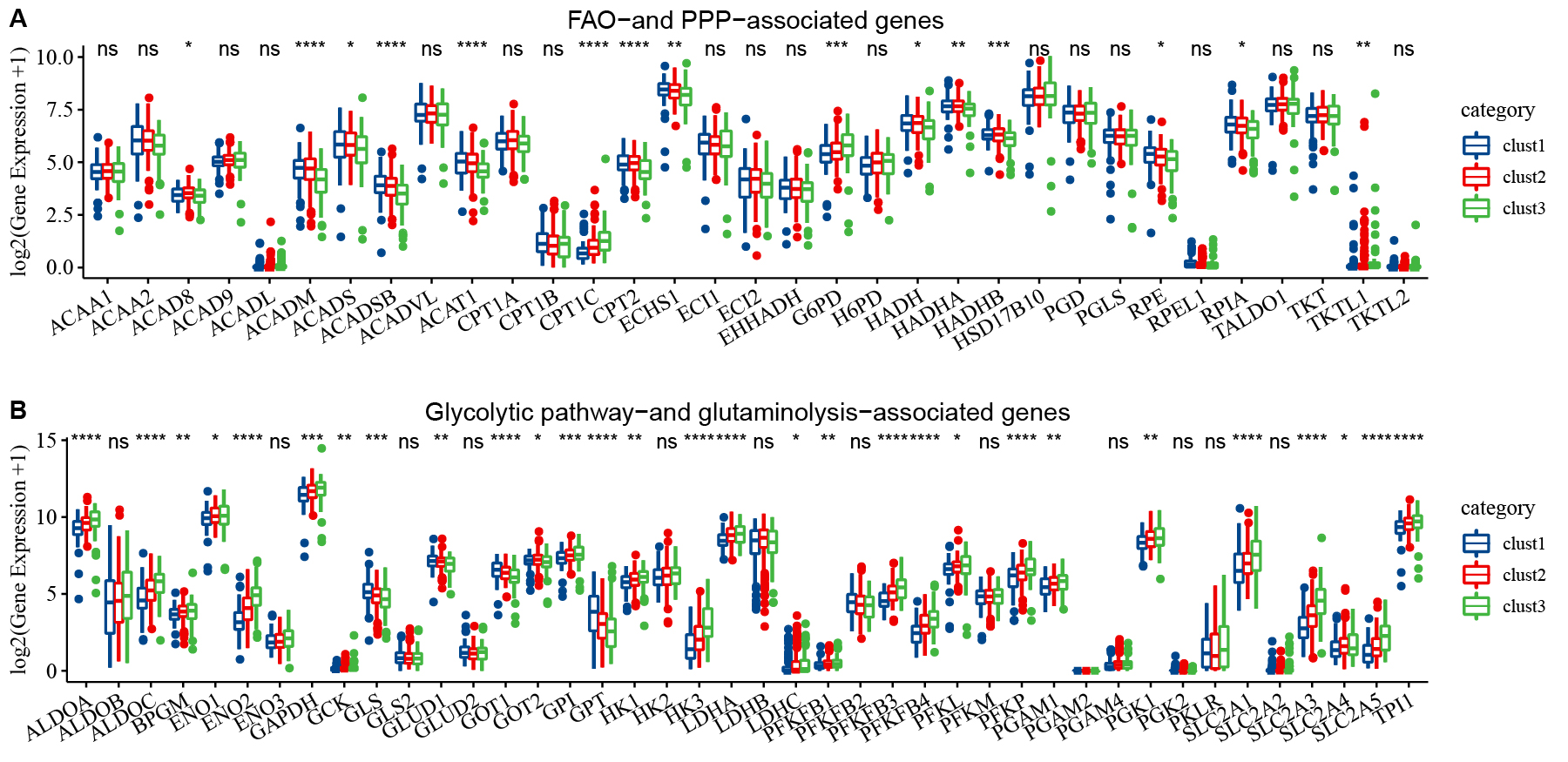

Supplement: Supplementary Materials — Supplementary Figure S1: the flowchart of this study. Supplementary Figure S2: expression of genes within four metabolism pathways in three clusters. Kruskal test was conducted. ns, no significance. ∗∗P < 0.01, ∗∗∗P < 0.001, and ∗∗∗∗P < 0.0001. Supplementary Figure S3: the distribution of three clusters in PCA plots. Supplementary Figure S4: the distribution of different clinical features in three clusters. Chi-square test was conducted. Supplementary Figure S5: significant CNVs detected by gistic2. Left line indicates chromosomes. Right line indicates significant CNVs (q value < 0.25). The bottom line indicates q values and the upper line indicates G score. Supplementary Figure S6: the enrichment score of immune cells in TCGA-COAD dataset analyzed by EPIC. Kruskal test was conducted. ns, no significant. ∗∗P < 0.01 and ∗∗∗∗P < 0.001. Supplementary Figure S7: TME features of three clusters in GSE17536 dataset. (a) Estimated proportion of 22 immune cells. (b) Stromal score, immune score, and ESTIMATE score calculated by ESTIMATE. (c) Enrichment score of 10 oncogenic pathways. ((d)–(h)) Enrichment scores of toll-like receptor, NK cytotoxicity, antigen processing and presentation, IFN-γ, and CYT. ANOVA was conducted. ns, no significance. ∗P < 0.05, ∗∗P < 0.01, ∗∗∗P < 0.01, and ∗∗∗∗P < 0.0001. Supplementary Figure S8: functional analysis on 69 genes within PPI network in TCGA-COAD dataset. ((a)–(c)) The top 10 significantly enriched terms in biological process (a), cellular component (b), and molecular function (c). (d) Nine significantly enriched KEGG pathways. Supplementary Figure S9: quality control and preprocessing of single-cell data. Supplementary Figure S10: the CNV heatmap of CopyKat for distinguishing malignant and nonmalignant cells. Orange indicates malignant cells and green indicates nonmalignant cells. Supplementary Figure S11: the ssGSEA enrichment score of hypoxia in three subtypes in TCGA-COAD dataset. Wilcoxon test was conducted. ∗∗∗∗P < 0.0001. Supplem [file 2159794.f1.zip › 2159794.f1/Supplementary Figure S2.jpg]

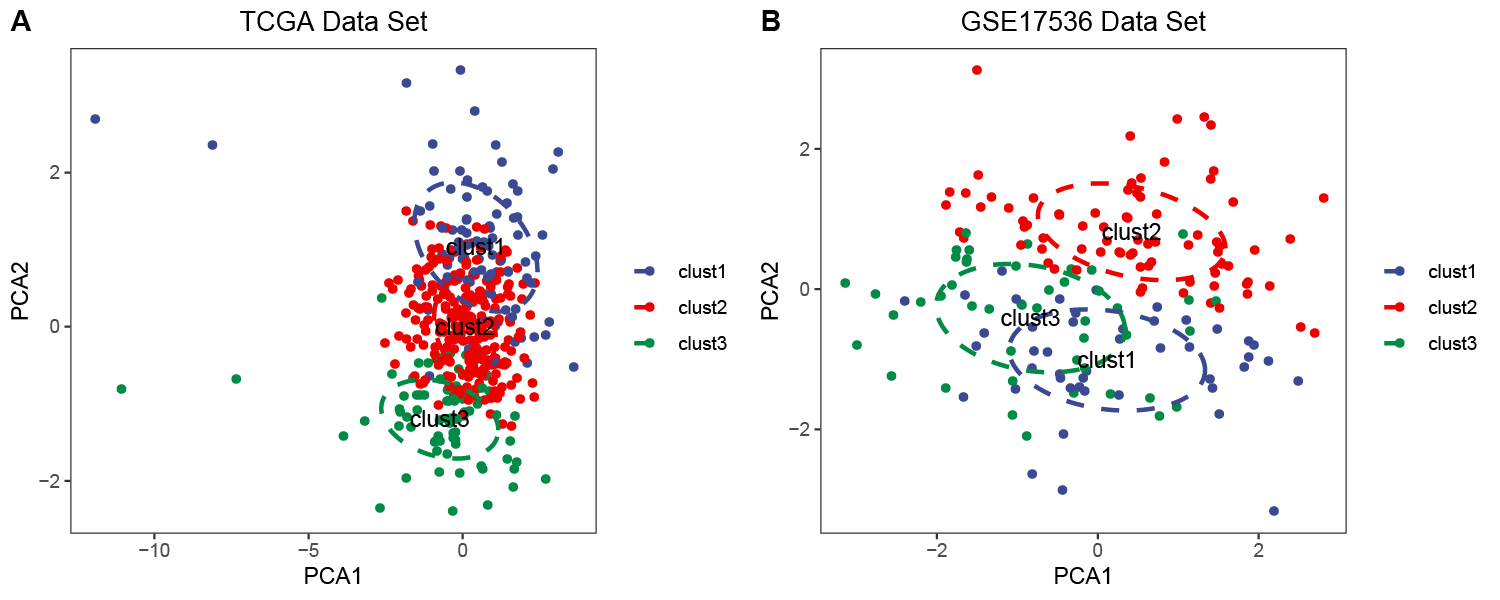

Supplement: Supplementary Materials — Supplementary Figure S1: the flowchart of this study. Supplementary Figure S2: expression of genes within four metabolism pathways in three clusters. Kruskal test was conducted. ns, no significance. ∗∗P < 0.01, ∗∗∗P < 0.001, and ∗∗∗∗P < 0.0001. Supplementary Figure S3: the distribution of three clusters in PCA plots. Supplementary Figure S4: the distribution of different clinical features in three clusters. Chi-square test was conducted. Supplementary Figure S5: significant CNVs detected by gistic2. Left line indicates chromosomes. Right line indicates significant CNVs (q value < 0.25). The bottom line indicates q values and the upper line indicates G score. Supplementary Figure S6: the enrichment score of immune cells in TCGA-COAD dataset analyzed by EPIC. Kruskal test was conducted. ns, no significant. ∗∗P < 0.01 and ∗∗∗∗P < 0.001. Supplementary Figure S7: TME features of three clusters in GSE17536 dataset. (a) Estimated proportion of 22 immune cells. (b) Stromal score, immune score, and ESTIMATE score calculated by ESTIMATE. (c) Enrichment score of 10 oncogenic pathways. ((d)–(h)) Enrichment scores of toll-like receptor, NK cytotoxicity, antigen processing and presentation, IFN-γ, and CYT. ANOVA was conducted. ns, no significance. ∗P < 0.05, ∗∗P < 0.01, ∗∗∗P < 0.01, and ∗∗∗∗P < 0.0001. Supplementary Figure S8: functional analysis on 69 genes within PPI network in TCGA-COAD dataset. ((a)–(c)) The top 10 significantly enriched terms in biological process (a), cellular component (b), and molecular function (c). (d) Nine significantly enriched KEGG pathways. Supplementary Figure S9: quality control and preprocessing of single-cell data. Supplementary Figure S10: the CNV heatmap of CopyKat for distinguishing malignant and nonmalignant cells. Orange indicates malignant cells and green indicates nonmalignant cells. Supplementary Figure S11: the ssGSEA enrichment score of hypoxia in three subtypes in TCGA-COAD dataset. Wilcoxon test was conducted. ∗∗∗∗P < 0.0001. Supplem [file 2159794.f1.zip › 2159794.f1/Supplementary Figure S3.jpg]

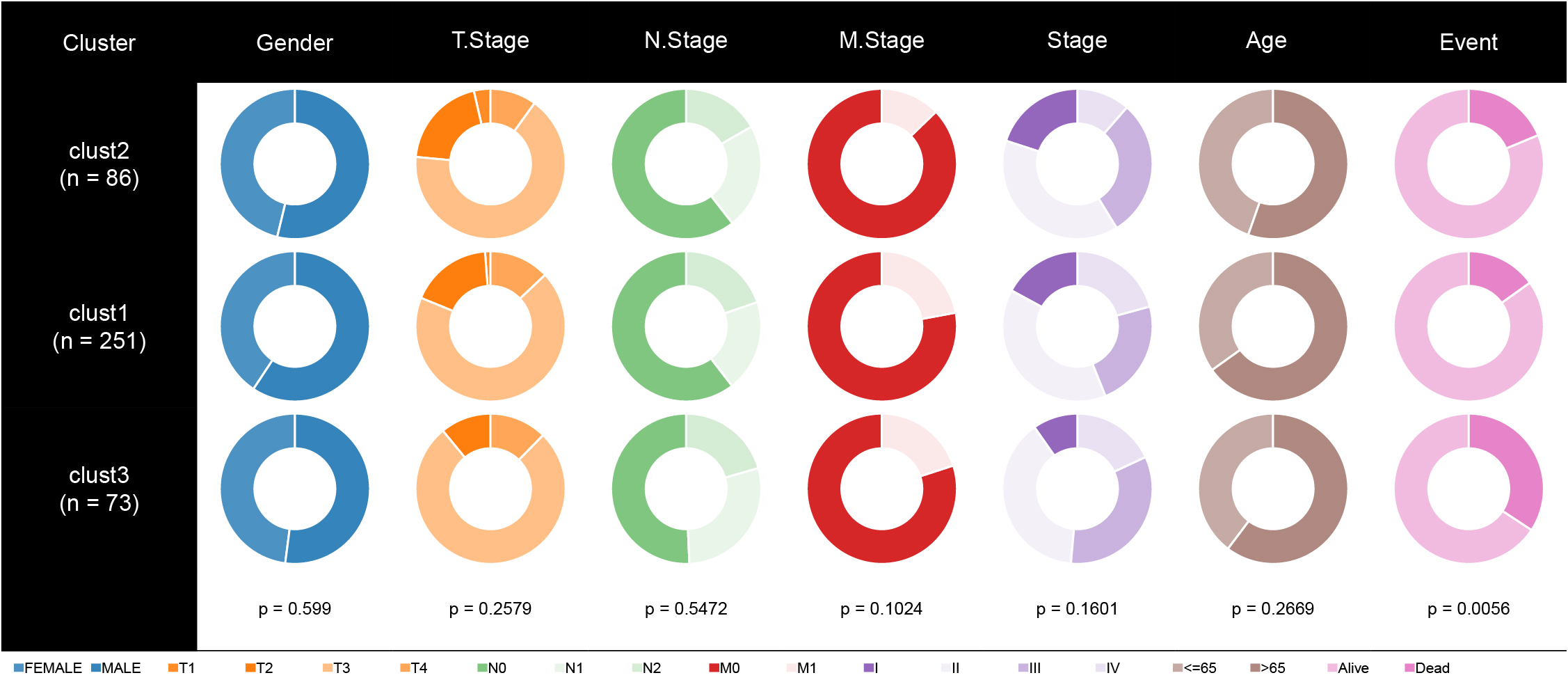

Supplement: Supplementary Materials — Supplementary Figure S1: the flowchart of this study. Supplementary Figure S2: expression of genes within four metabolism pathways in three clusters. Kruskal test was conducted. ns, no significance. ∗∗P < 0.01, ∗∗∗P < 0.001, and ∗∗∗∗P < 0.0001. Supplementary Figure S3: the distribution of three clusters in PCA plots. Supplementary Figure S4: the distribution of different clinical features in three clusters. Chi-square test was conducted. Supplementary Figure S5: significant CNVs detected by gistic2. Left line indicates chromosomes. Right line indicates significant CNVs (q value < 0.25). The bottom line indicates q values and the upper line indicates G score. Supplementary Figure S6: the enrichment score of immune cells in TCGA-COAD dataset analyzed by EPIC. Kruskal test was conducted. ns, no significant. ∗∗P < 0.01 and ∗∗∗∗P < 0.001. Supplementary Figure S7: TME features of three clusters in GSE17536 dataset. (a) Estimated proportion of 22 immune cells. (b) Stromal score, immune score, and ESTIMATE score calculated by ESTIMATE. (c) Enrichment score of 10 oncogenic pathways. ((d)–(h)) Enrichment scores of toll-like receptor, NK cytotoxicity, antigen processing and presentation, IFN-γ, and CYT. ANOVA was conducted. ns, no significance. ∗P < 0.05, ∗∗P < 0.01, ∗∗∗P < 0.01, and ∗∗∗∗P < 0.0001. Supplementary Figure S8: functional analysis on 69 genes within PPI network in TCGA-COAD dataset. ((a)–(c)) The top 10 significantly enriched terms in biological process (a), cellular component (b), and molecular function (c). (d) Nine significantly enriched KEGG pathways. Supplementary Figure S9: quality control and preprocessing of single-cell data. Supplementary Figure S10: the CNV heatmap of CopyKat for distinguishing malignant and nonmalignant cells. Orange indicates malignant cells and green indicates nonmalignant cells. Supplementary Figure S11: the ssGSEA enrichment score of hypoxia in three subtypes in TCGA-COAD dataset. Wilcoxon test was conducted. ∗∗∗∗P < 0.0001. Supplem [file 2159794.f1.zip › 2159794.f1/Supplementary Figure S4.jpg]

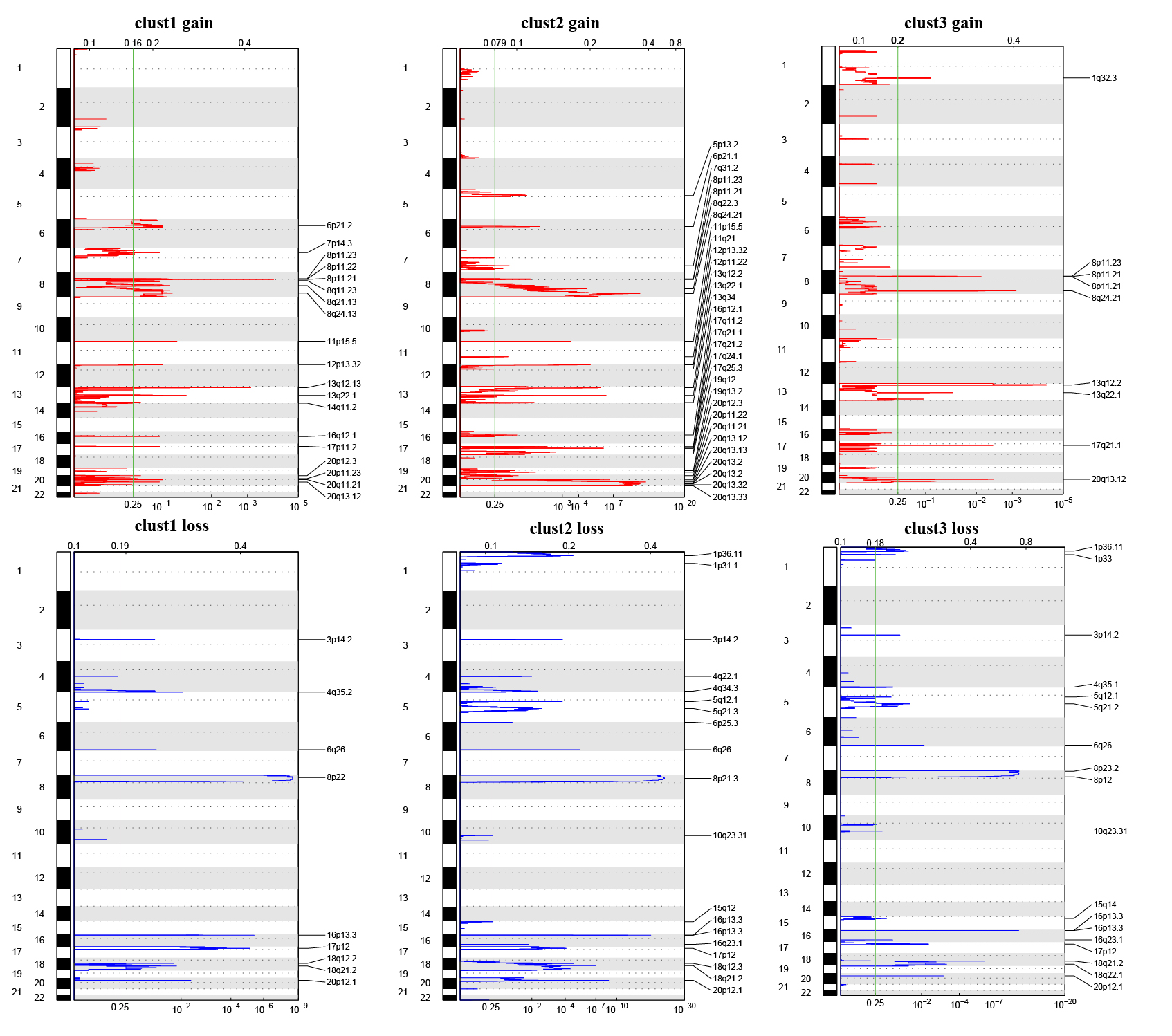

Supplement: Supplementary Materials — Supplementary Figure S1: the flowchart of this study. Supplementary Figure S2: expression of genes within four metabolism pathways in three clusters. Kruskal test was conducted. ns, no significance. ∗∗P < 0.01, ∗∗∗P < 0.001, and ∗∗∗∗P < 0.0001. Supplementary Figure S3: the distribution of three clusters in PCA plots. Supplementary Figure S4: the distribution of different clinical features in three clusters. Chi-square test was conducted. Supplementary Figure S5: significant CNVs detected by gistic2. Left line indicates chromosomes. Right line indicates significant CNVs (q value < 0.25). The bottom line indicates q values and the upper line indicates G score. Supplementary Figure S6: the enrichment score of immune cells in TCGA-COAD dataset analyzed by EPIC. Kruskal test was conducted. ns, no significant. ∗∗P < 0.01 and ∗∗∗∗P < 0.001. Supplementary Figure S7: TME features of three clusters in GSE17536 dataset. (a) Estimated proportion of 22 immune cells. (b) Stromal score, immune score, and ESTIMATE score calculated by ESTIMATE. (c) Enrichment score of 10 oncogenic pathways. ((d)–(h)) Enrichment scores of toll-like receptor, NK cytotoxicity, antigen processing and presentation, IFN-γ, and CYT. ANOVA was conducted. ns, no significance. ∗P < 0.05, ∗∗P < 0.01, ∗∗∗P < 0.01, and ∗∗∗∗P < 0.0001. Supplementary Figure S8: functional analysis on 69 genes within PPI network in TCGA-COAD dataset. ((a)–(c)) The top 10 significantly enriched terms in biological process (a), cellular component (b), and molecular function (c). (d) Nine significantly enriched KEGG pathways. Supplementary Figure S9: quality control and preprocessing of single-cell data. Supplementary Figure S10: the CNV heatmap of CopyKat for distinguishing malignant and nonmalignant cells. Orange indicates malignant cells and green indicates nonmalignant cells. Supplementary Figure S11: the ssGSEA enrichment score of hypoxia in three subtypes in TCGA-COAD dataset. Wilcoxon test was conducted. ∗∗∗∗P < 0.0001. Supplem [file 2159794.f1.zip › 2159794.f1/Supplementary Figure S5.jpg]

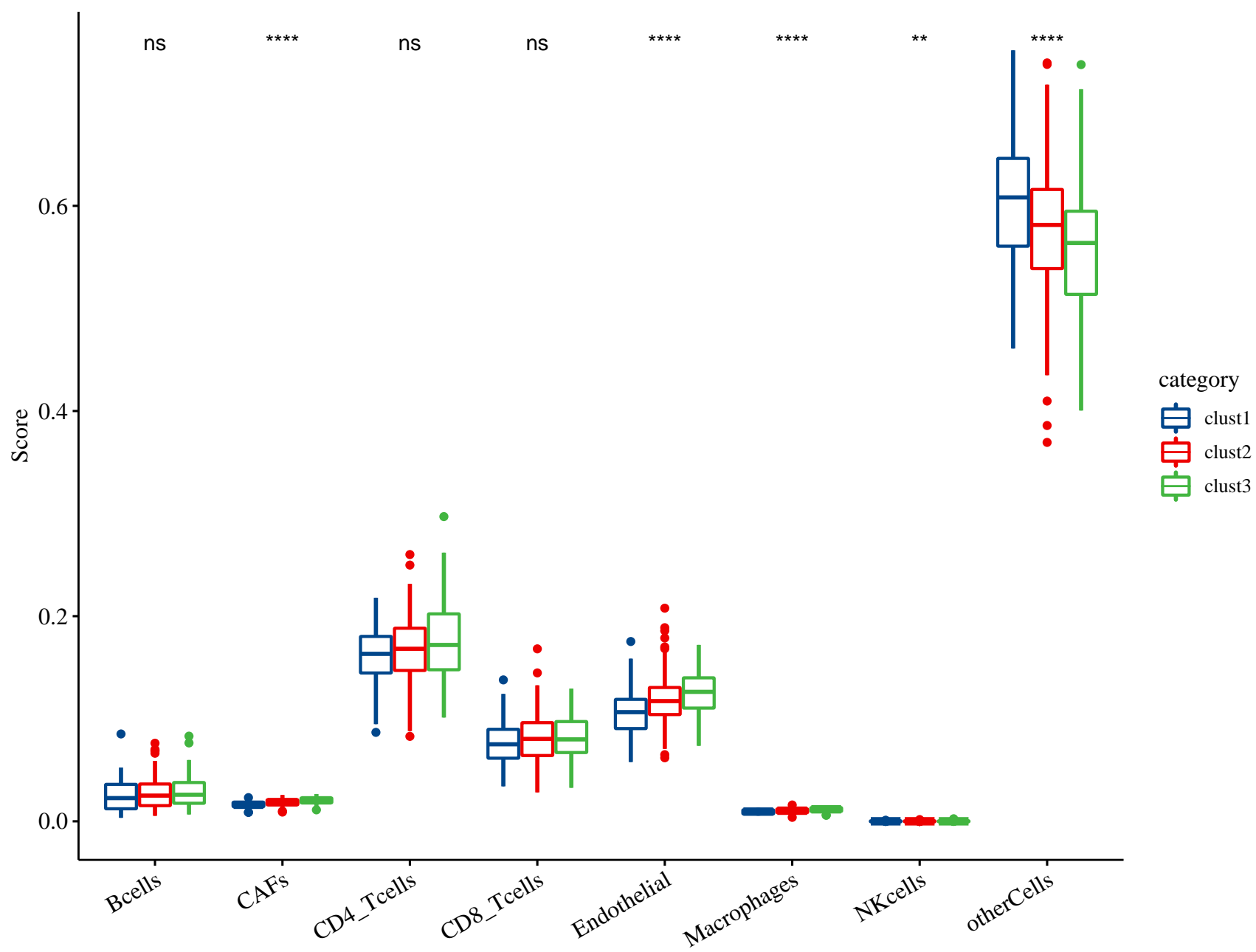

Supplement: Supplementary Materials — Supplementary Figure S1: the flowchart of this study. Supplementary Figure S2: expression of genes within four metabolism pathways in three clusters. Kruskal test was conducted. ns, no significance. ∗∗P < 0.01, ∗∗∗P < 0.001, and ∗∗∗∗P < 0.0001. Supplementary Figure S3: the distribution of three clusters in PCA plots. Supplementary Figure S4: the distribution of different clinical features in three clusters. Chi-square test was conducted. Supplementary Figure S5: significant CNVs detected by gistic2. Left line indicates chromosomes. Right line indicates significant CNVs (q value < 0.25). The bottom line indicates q values and the upper line indicates G score. Supplementary Figure S6: the enrichment score of immune cells in TCGA-COAD dataset analyzed by EPIC. Kruskal test was conducted. ns, no significant. ∗∗P < 0.01 and ∗∗∗∗P < 0.001. Supplementary Figure S7: TME features of three clusters in GSE17536 dataset. (a) Estimated proportion of 22 immune cells. (b) Stromal score, immune score, and ESTIMATE score calculated by ESTIMATE. (c) Enrichment score of 10 oncogenic pathways. ((d)–(h)) Enrichment scores of toll-like receptor, NK cytotoxicity, antigen processing and presentation, IFN-γ, and CYT. ANOVA was conducted. ns, no significance. ∗P < 0.05, ∗∗P < 0.01, ∗∗∗P < 0.01, and ∗∗∗∗P < 0.0001. Supplementary Figure S8: functional analysis on 69 genes within PPI network in TCGA-COAD dataset. ((a)–(c)) The top 10 significantly enriched terms in biological process (a), cellular component (b), and molecular function (c). (d) Nine significantly enriched KEGG pathways. Supplementary Figure S9: quality control and preprocessing of single-cell data. Supplementary Figure S10: the CNV heatmap of CopyKat for distinguishing malignant and nonmalignant cells. Orange indicates malignant cells and green indicates nonmalignant cells. Supplementary Figure S11: the ssGSEA enrichment score of hypoxia in three subtypes in TCGA-COAD dataset. Wilcoxon test was conducted. ∗∗∗∗P < 0.0001. Supplem [file 2159794.f1.zip › 2159794.f1/Supplementary Figure S6.pdf]

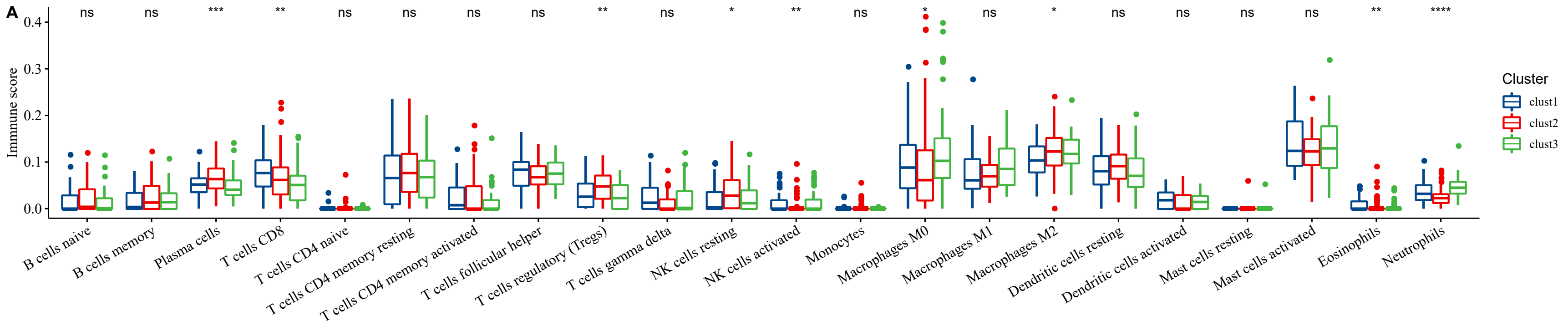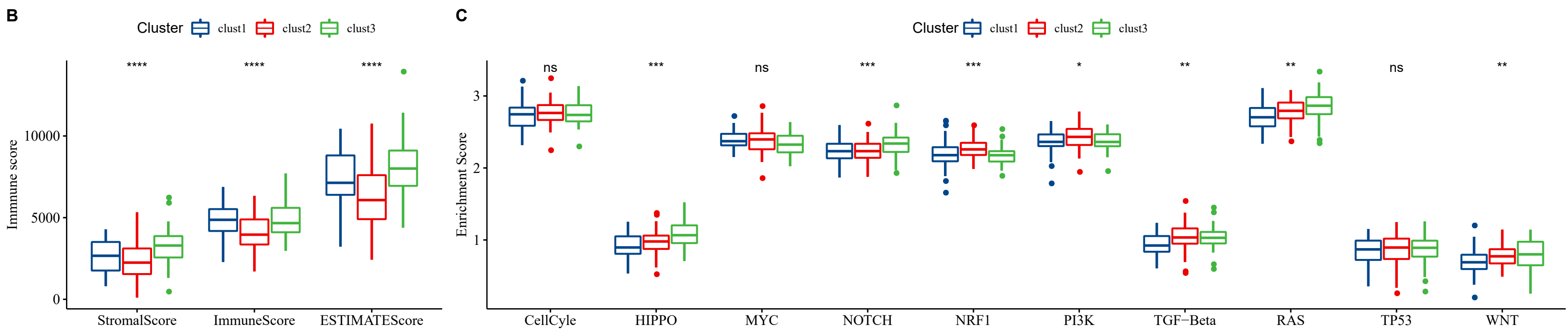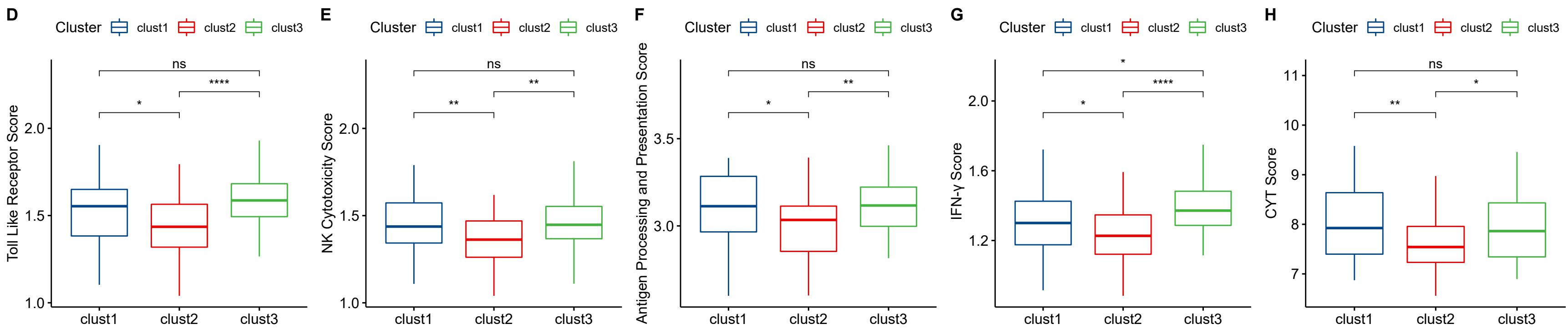

Supplement: Supplementary Materials — Supplementary Figure S1: the flowchart of this study. Supplementary Figure S2: expression of genes within four metabolism pathways in three clusters. Kruskal test was conducted. ns, no significance. ∗∗P < 0.01, ∗∗∗P < 0.001, and ∗∗∗∗P < 0.0001. Supplementary Figure S3: the distribution of three clusters in PCA plots. Supplementary Figure S4: the distribution of different clinical features in three clusters. Chi-square test was conducted. Supplementary Figure S5: significant CNVs detected by gistic2. Left line indicates chromosomes. Right line indicates significant CNVs (q value < 0.25). The bottom line indicates q values and the upper line indicates G score. Supplementary Figure S6: the enrichment score of immune cells in TCGA-COAD dataset analyzed by EPIC. Kruskal test was conducted. ns, no significant. ∗∗P < 0.01 and ∗∗∗∗P < 0.001. Supplementary Figure S7: TME features of three clusters in GSE17536 dataset. (a) Estimated proportion of 22 immune cells. (b) Stromal score, immune score, and ESTIMATE score calculated by ESTIMATE. (c) Enrichment score of 10 oncogenic pathways. ((d)–(h)) Enrichment scores of toll-like receptor, NK cytotoxicity, antigen processing and presentation, IFN-γ, and CYT. ANOVA was conducted. ns, no significance. ∗P < 0.05, ∗∗P < 0.01, ∗∗∗P < 0.01, and ∗∗∗∗P < 0.0001. Supplementary Figure S8: functional analysis on 69 genes within PPI network in TCGA-COAD dataset. ((a)–(c)) The top 10 significantly enriched terms in biological process (a), cellular component (b), and molecular function (c). (d) Nine significantly enriched KEGG pathways. Supplementary Figure S9: quality control and preprocessing of single-cell data. Supplementary Figure S10: the CNV heatmap of CopyKat for distinguishing malignant and nonmalignant cells. Orange indicates malignant cells and green indicates nonmalignant cells. Supplementary Figure S11: the ssGSEA enrichment score of hypoxia in three subtypes in TCGA-COAD dataset. Wilcoxon test was conducted. ∗∗∗∗P < 0.0001. Supplem [file 2159794.f1.zip › 2159794.f1/Supplementary Figure S7.pdf]

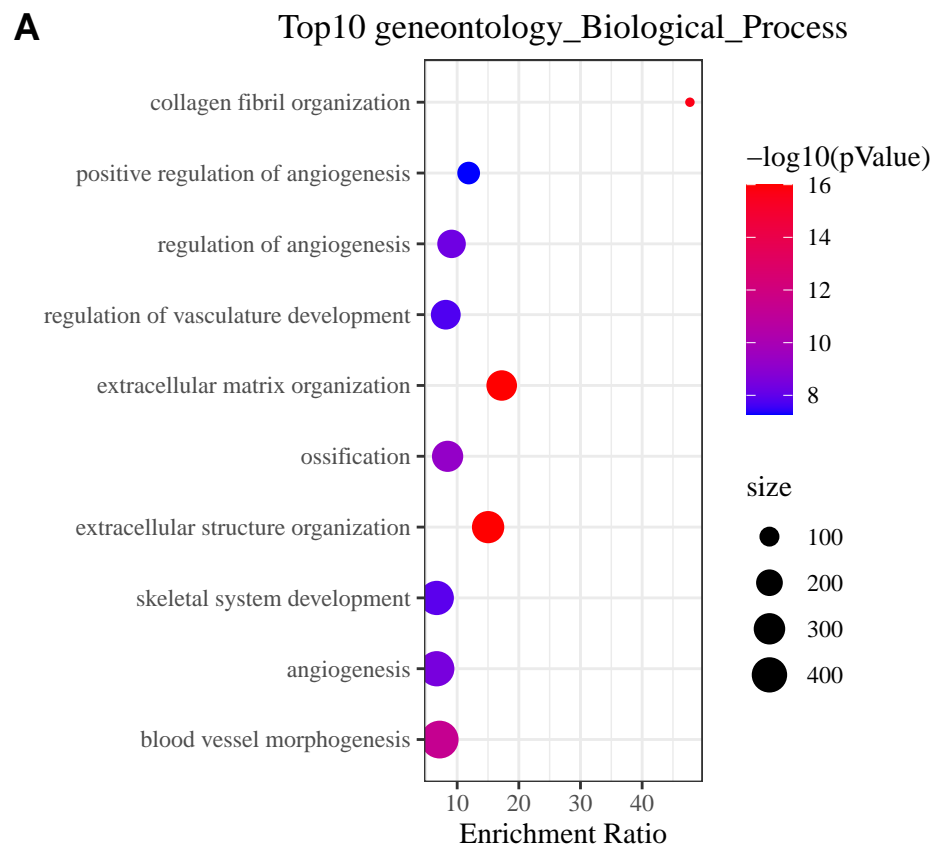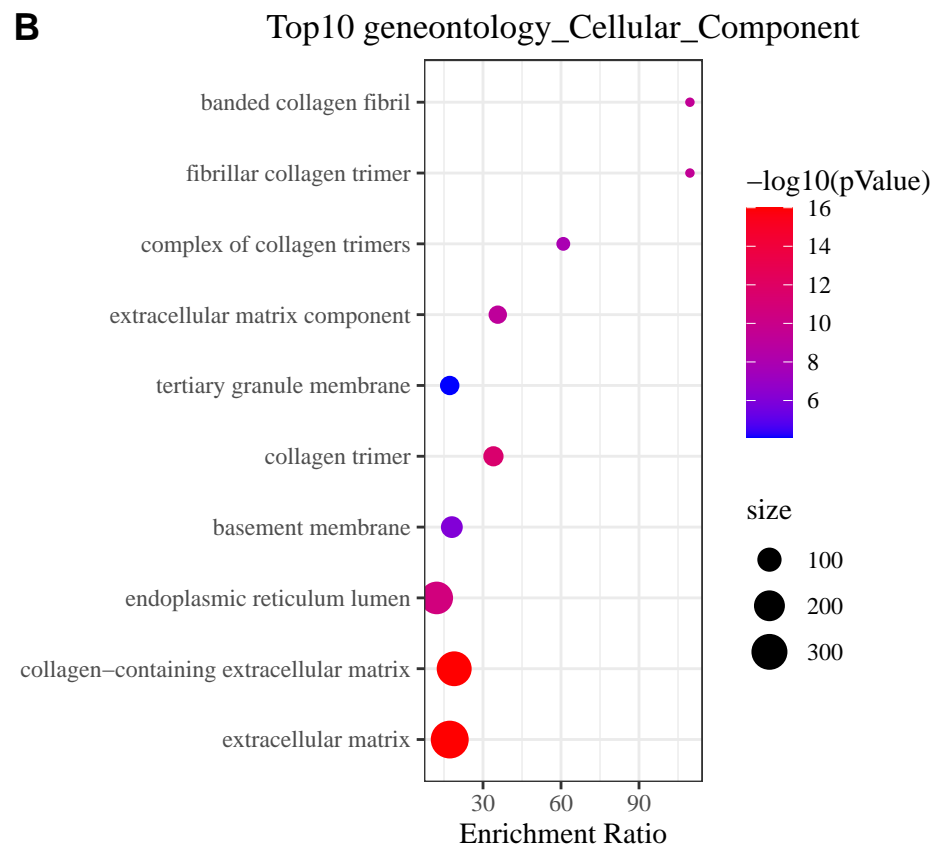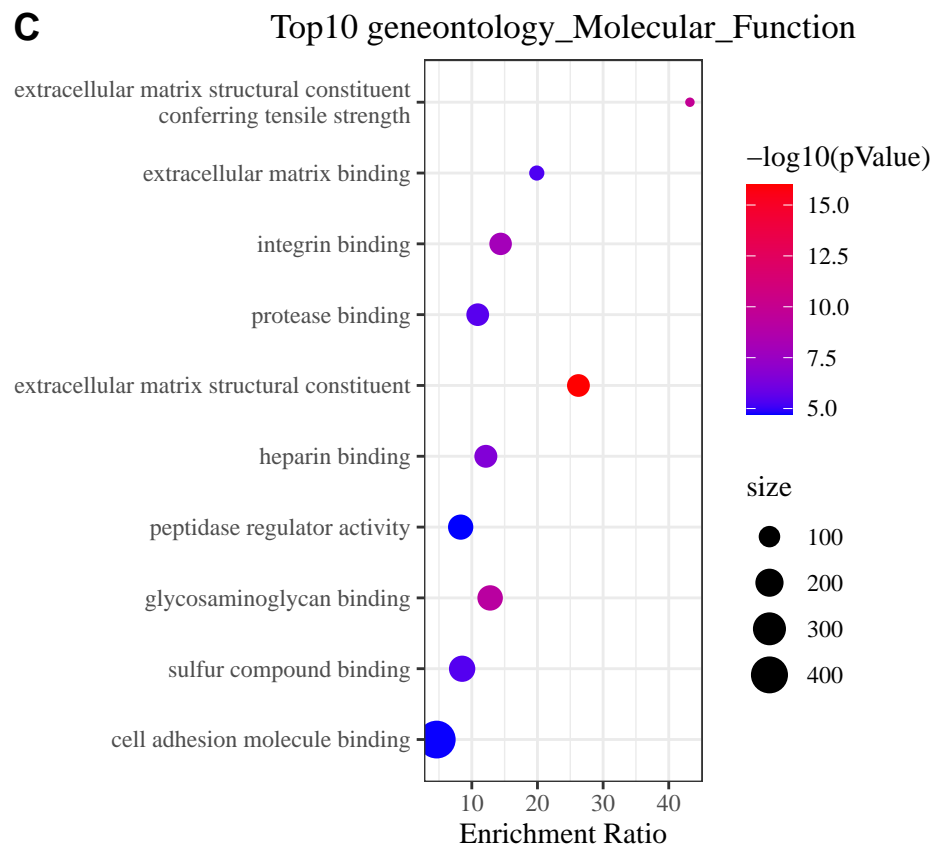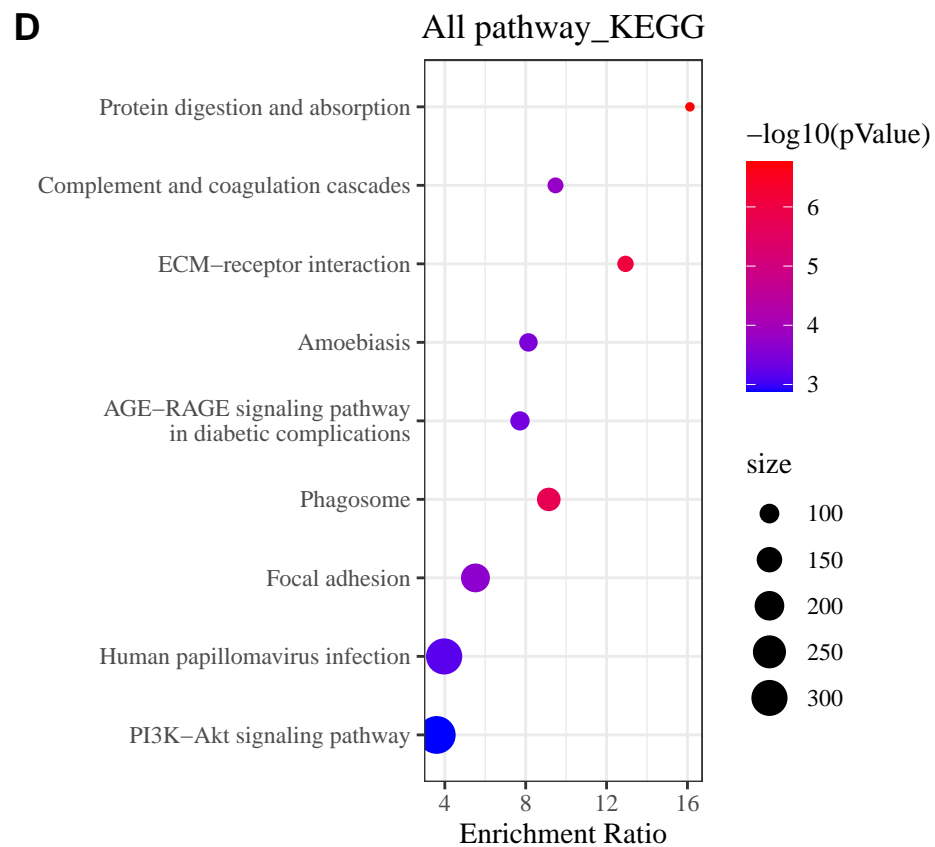

Supplement: Supplementary Materials — Supplementary Figure S1: the flowchart of this study. Supplementary Figure S2: expression of genes within four metabolism pathways in three clusters. Kruskal test was conducted. ns, no significance. ∗∗P < 0.01, ∗∗∗P < 0.001, and ∗∗∗∗P < 0.0001. Supplementary Figure S3: the distribution of three clusters in PCA plots. Supplementary Figure S4: the distribution of different clinical features in three clusters. Chi-square test was conducted. Supplementary Figure S5: significant CNVs detected by gistic2. Left line indicates chromosomes. Right line indicates significant CNVs (q value < 0.25). The bottom line indicates q values and the upper line indicates G score. Supplementary Figure S6: the enrichment score of immune cells in TCGA-COAD dataset analyzed by EPIC. Kruskal test was conducted. ns, no significant. ∗∗P < 0.01 and ∗∗∗∗P < 0.001. Supplementary Figure S7: TME features of three clusters in GSE17536 dataset. (a) Estimated proportion of 22 immune cells. (b) Stromal score, immune score, and ESTIMATE score calculated by ESTIMATE. (c) Enrichment score of 10 oncogenic pathways. ((d)–(h)) Enrichment scores of toll-like receptor, NK cytotoxicity, antigen processing and presentation, IFN-γ, and CYT. ANOVA was conducted. ns, no significance. ∗P < 0.05, ∗∗P < 0.01, ∗∗∗P < 0.01, and ∗∗∗∗P < 0.0001. Supplementary Figure S8: functional analysis on 69 genes within PPI network in TCGA-COAD dataset. ((a)–(c)) The top 10 significantly enriched terms in biological process (a), cellular component (b), and molecular function (c). (d) Nine significantly enriched KEGG pathways. Supplementary Figure S9: quality control and preprocessing of single-cell data. Supplementary Figure S10: the CNV heatmap of CopyKat for distinguishing malignant and nonmalignant cells. Orange indicates malignant cells and green indicates nonmalignant cells. Supplementary Figure S11: the ssGSEA enrichment score of hypoxia in three subtypes in TCGA-COAD dataset. Wilcoxon test was conducted. ∗∗∗∗P < 0.0001. Supplem [file 2159794.f1.zip › 2159794.f1/Supplementary Figure S8.pdf]

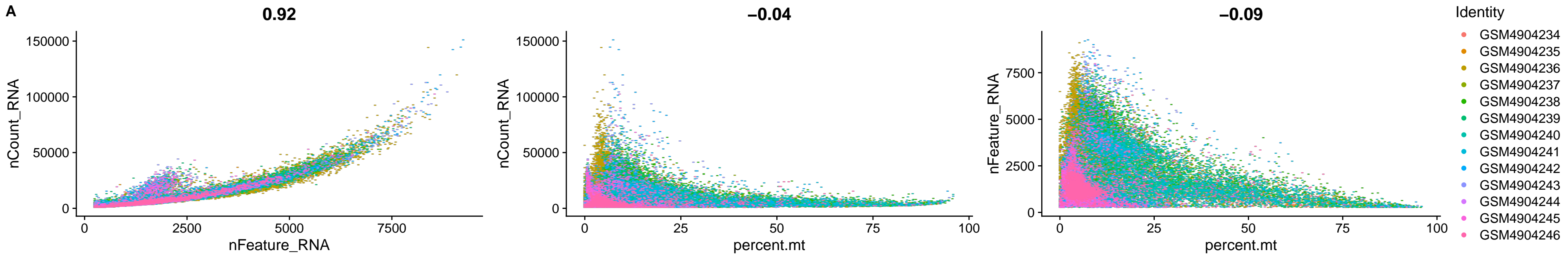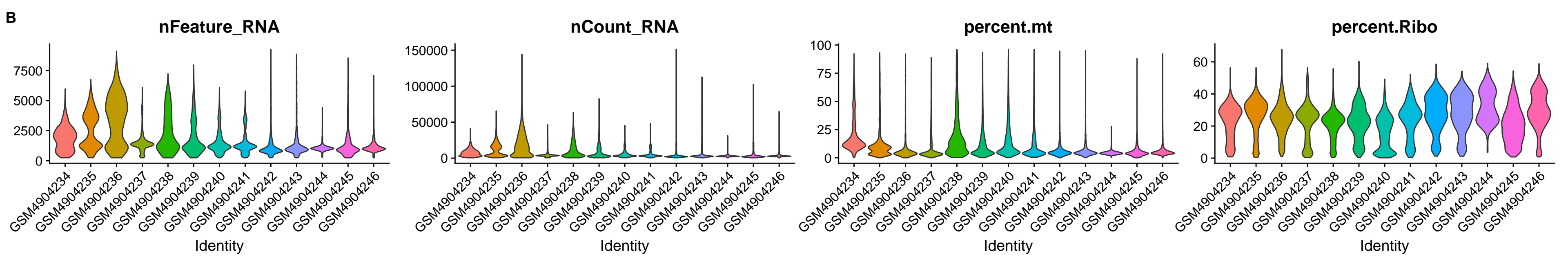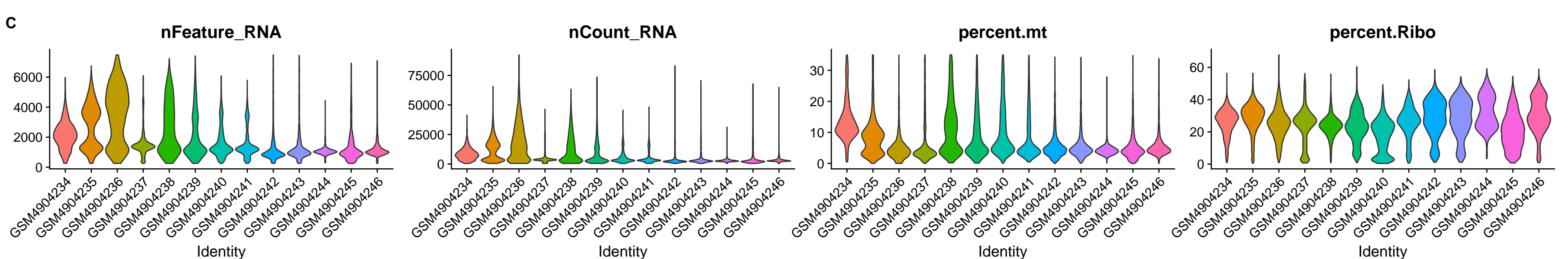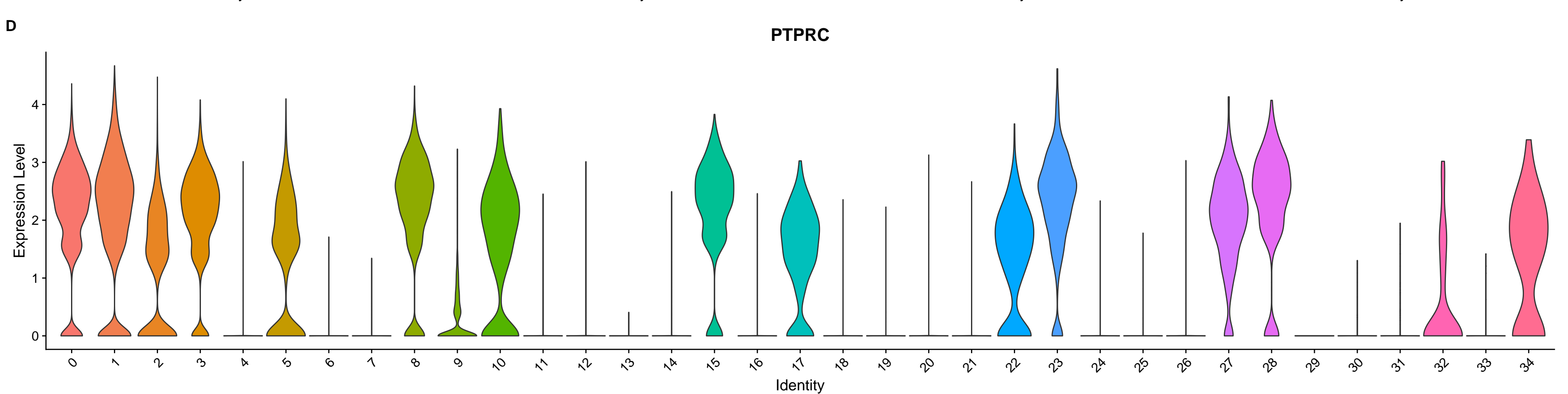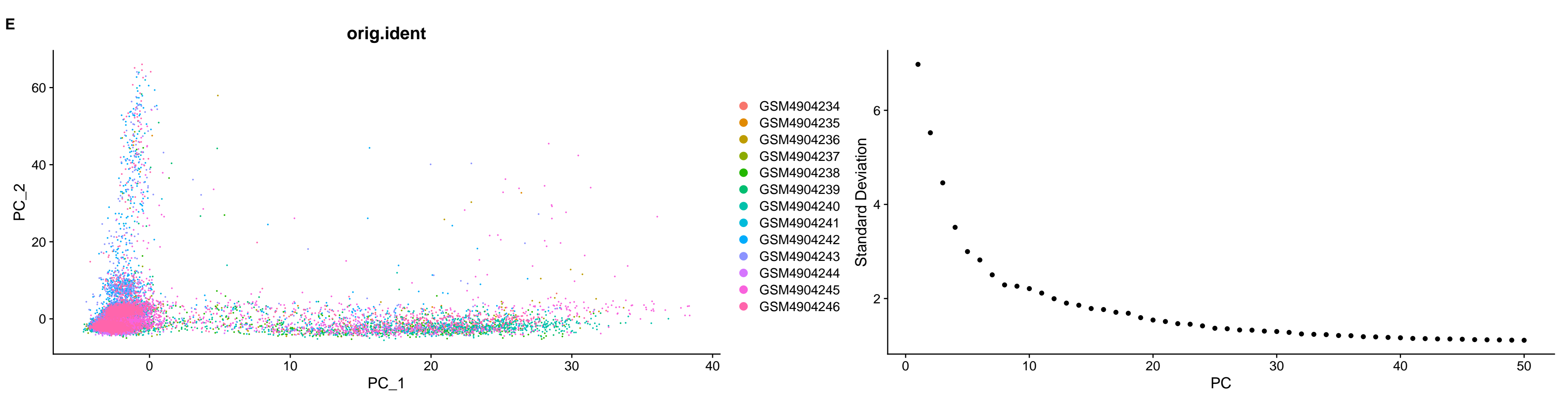

Supplement: Supplementary Materials — Supplementary Figure S1: the flowchart of this study. Supplementary Figure S2: expression of genes within four metabolism pathways in three clusters. Kruskal test was conducted. ns, no significance. ∗∗P < 0.01, ∗∗∗P < 0.001, and ∗∗∗∗P < 0.0001. Supplementary Figure S3: the distribution of three clusters in PCA plots. Supplementary Figure S4: the distribution of different clinical features in three clusters. Chi-square test was conducted. Supplementary Figure S5: significant CNVs detected by gistic2. Left line indicates chromosomes. Right line indicates significant CNVs (q value < 0.25). The bottom line indicates q values and the upper line indicates G score. Supplementary Figure S6: the enrichment score of immune cells in TCGA-COAD dataset analyzed by EPIC. Kruskal test was conducted. ns, no significant. ∗∗P < 0.01 and ∗∗∗∗P < 0.001. Supplementary Figure S7: TME features of three clusters in GSE17536 dataset. (a) Estimated proportion of 22 immune cells. (b) Stromal score, immune score, and ESTIMATE score calculated by ESTIMATE. (c) Enrichment score of 10 oncogenic pathways. ((d)–(h)) Enrichment scores of toll-like receptor, NK cytotoxicity, antigen processing and presentation, IFN-γ, and CYT. ANOVA was conducted. ns, no significance. ∗P < 0.05, ∗∗P < 0.01, ∗∗∗P < 0.01, and ∗∗∗∗P < 0.0001. Supplementary Figure S8: functional analysis on 69 genes within PPI network in TCGA-COAD dataset. ((a)–(c)) The top 10 significantly enriched terms in biological process (a), cellular component (b), and molecular function (c). (d) Nine significantly enriched KEGG pathways. Supplementary Figure S9: quality control and preprocessing of single-cell data. Supplementary Figure S10: the CNV heatmap of CopyKat for distinguishing malignant and nonmalignant cells. Orange indicates malignant cells and green indicates nonmalignant cells. Supplementary Figure S11: the ssGSEA enrichment score of hypoxia in three subtypes in TCGA-COAD dataset. Wilcoxon test was conducted. ∗∗∗∗P < 0.0001. Supplem [file 2159794.f1.zip › 2159794.f1/Supplementary Figure S9.pdf]
